# Artificial intelligence-based prediction of diseases among homeless populations in Bogotá: Implications for targeted interventions

**DOI:** 10.1371/journal.pone.0352268

**Published:** 2026-06-26

**Authors:** Hugo Ordoñez, Cristian Camilo Ordoñez, Juan-Sebastian Gonzalez-Sanabria

**Affiliations:** 1 Universidad del Cauca, Popayán, Cauca, Colombia; 2 Universidad Cooperativa de Colombia, Pasto, Colombia; 3 Universidad Pedagógica y Tecnológica de Colombia, Tunja, Colombia; Norbert Wiener University, PERU

## Abstract

**Background:**

The homeless population in Bogotá exhibits complex social and health vulnerabilities, with a high prevalence of chronic and communicable diseases such as hypertension, diabetes, tuberculosis, HIV/AIDS, and cancer. These conditions hinder epidemiological surveillance and timely public health decision-making, particularly in contexts of social exclusion.

**Objective:**

To develop and evaluate an artificial intelligence-based predictive model aimed at identifying disease occurrence risk profiles among the homeless population in Bogotá, in order to support public health decision-making.

**Methods:**

Data from the 2024 Bogotá Homeless Census were analyzed, comprising 10 478 records and 46 demographic, clinical, and socioeconomic variables. Data processing and model development followed the CRISP-DM methodology. The extreme gradient boosting (XGBoost) algorithm was implemented to predict disease occurrence. Model performance was evaluated in terms of accuracy, sensitivity, and the F1-score, while interpretability was assessed through SHAP (SHapley Additive exPlanations) values. Additionally, metrics related to social trust, system response time, and potential health impacts were examined.

**Results:**

The model achieved an accuracy of 0.91, a sensitivity of 0.57, and an F1-score of 0.70, striking an adequate balance between identifying high-risk individuals and reducing false positives. SHAP analysis identified hypertension, diabetes, and HIV/AIDS as the main predictors of classification. However, complementary metrics revealed limitations in social trust (TAS = 0.49), system response time (SRT = 24.86 hours), and potential health impact (PHIS = 0.24).

**Conclusions and implications:**

These findings suggest that explainable artificial intelligence (XAI) models may support public health surveillance and intervention prioritization in homeless populations by identifying epidemiological risk profiles. However, the models’ applicability remains context-specific and requires external validation before implementation across other vulnerable populations or healthcare settings.

## Introduction

The phenomenon of homelessness in Bogotá (Colombia) is the result of a complex interaction of structural and social factors [1]. Historical socioeconomic inequality, characterized by limited access to education, employment, and adequate housing, has perpetuated cycles of urban poverty and vulnerability [2]. Forced displacement due to the country’s internal armed conflict has driven thousands of people to Bogotá, many of whom arrive without social support networks, increasing their risk of becoming homeless [3]. Likewise, internal and cross-border migration, especially from Venezuela, has intensified the city’s social challenges, as migrants often face legal, economic, and social barriers to integration [4]. The breakdown of family and community ties, often worsened by domestic violence, abuse, or abandonment, further increases the likelihood of homelessness. Additionally, untreated mental health conditions and substance abuse play a critical role both in entering and remaining in street life [5].

Despite the existence of public programs, the lack of comprehensive and sustainable social inclusion policies limits the effectiveness of interventions. Furthermore, persistent stigma and discrimination towards homeless individuals deepen their marginalization, hindering access to essential services such as healthcare, social support, and employment. This situation is significantly worsened by the high prevalence of chronic and communicable diseases among this population, including hypertension, cancer, tuberculosis, HIV/AIDS, and diabetes [6,7]. Living under extreme conditions, lacking regular medical check-ups, and having limited access to timely treatment increase the risk of complications and premature mortality. Moreover, poor continuity in treatment regimens and low adherence to medications contribute to disease progression, affecting individual and public health in the city [8].

The challenges faced by homeless populations and their associated health conditions have attracted increasing scientific interest due to their implications for public health, social security, and urban planning. In this context, artificial intelligence (AI) has emerged as a key tool for developing decision support systems that identify risk patterns, predict disease progression, and optimize personalized interventions. Several studies have shown that machine learning models and neural networks can enable the early detection of chronic and infectious diseases such as diabetes, tuberculosis, and HIV/AIDS in vulnerable populations [9,10]. These systems aid health authorities and professionals in prioritizing critical cases, efficiently allocating resources, and designing more inclusive public policies. Other works have used classification algorithms (*e.g.*, random forests, XGBoost, and deep neural networks) to model risk factors and predict medical conditions in homeless individuals [11]. Furthermore, AI-based initiatives have enabled the integration of clinical, social, and demographic data to build risk profiles and suggest individualized interventions, enhancing the effectiveness of care and reintegration programs [12,13].

Although artificial intelligence has been increasingly applied to the study of homeless populations, most empirical work has been conducted in high-income countries, particularly in the United States and the United Kingdom [14,15–20]. This geographical concentration highlights the existence of an “AI gap,” given that the proposed models have been designed and validated in health systems characterized by high administrative integration, broad insurance coverage, and the availability of interoperable databases, conditions typical of developed countries [14,18].

This scenario contrasts significantly with the Latin American context. In Colombia, for example, the health system is highly fragmented in terms of service delivery and there are barriers to continuity of care, especially for highly vulnerable populations such as homeless people [6,8]. This reality is also influenced by structural dynamics specific to the region, including forced displacement, cross-border migration [3,4], and high levels of informal employment, factors that shape epidemiological patterns and trajectories of vulnerability that differ from those observed in high-income countries.

Consequently, models developed in the Global North cannot be assumed to be directly transferable without rigorous validation and contextual adaptation processes [17,18]. Their uncritical application could compromise the external validity of the results and reproduce pre-existing structural biases. Overcoming this gap requires the development of locally contextualized models that are explainable and tailored to the institutional, social, and epidemiological realities of middle-income countries.

In light of the above, this study presents an AI-based model for predicting diseases in the homeless population of Bogotá. The model seeks to support decision-making in governmental programs aimed at addressing the multiple health needs of the homeless population. These include initiatives led by the District Health Secretariat, such as *MAS Bienestar* model and the *HEARTS* program, which focus on hypertension screening through free check-ups at public events (like *Calles Abiertas*) and community-based primary care [21]. The city also hosts the *District Tuberculosis Control Program*, which is grounded in Resolution 227 of 2020 and promotes active case finding and comprehensive follow-up, especially in vulnerable populations such as the homeless [22]. At the national level, the Social Policy for Homeless People (PPSHC 2022–2031) establishes intersectoral care pathways to ensure adherence to tuberculosis and HIV/AIDS treatments and improve access to healthcare [23]. These initiatives combine medical, psychosocial, and community strategies that can be strengthened through AI-based decision support systems.

To build the proposed model, data from the 2024 Bogotá Homeless Census were used [24], which provides statistical information on the homeless population, including demographic characteristics, income sources (*e.g.*, recycling), and causes of homelessness (such as substance use), among other factors. These data are available through the DANE Open Data Portal. They are publicly accessible, anonymized, and available for research use by the general public. Therefore, no additional permissions were required for their use. The study was based on a secondary analysis of official information, with no direct contact with individuals and no use of identifiable data. There were no risks to the privacy or rights of the censused population. Consequently, individual informed consent and approval by an ethics committee were not required, as the data were used exclusively for scientific and public interest purposes, in accordance with ethical principles of confidentiality and responsible data use. The dataset consisted of 10,478 records and 46 variables.

The model’s evaluation results reveal significant trends related to the number of years spent living on the streets and the most prevalent diseases within this population. The analysis also uncovers the main reasons why individuals become homeless, the most commonly used psychoactive substances, the strategies employed to access healthcare, and the mechanisms used to obtain daily sustenance. Our findings, grounded in quantitative analysis, represent a valuable tool for supporting decision-making in intervention programs and public policies implemented by the aforementioned district and local authorities.

As previously mentioned, the purpose of this study was to develop and evaluate a machine learning-based predictive model to identify disease risk profiles among the homeless population of Bogotá, using data from the 2024 Homeless Census. Specifically, the study pursued the following objectives: (i) to assess, from an epidemiological perspective, machine learning models’ ability to identify patterns and factors associated with the occurrence of chronic and communicable diseases for the purposes of public health surveillance; (ii) to analyze, from an ethical and responsible AI perspective, our model’s performance, interpretability, and social acceptability, considering its potential impact in contexts of high vulnerability; and, (iii) to explore, from a public policy perspective, the use of our model as a decision-support tool for prioritizing interventions and efficiently allocating resources in public health programs targeting the homeless population of Bogotá.

## Background

AI applications aimed at the homeless have made significant progress through predictive models, recommendation systems, and prioritization tools. These studies provide solid evidence to implement AI-based solutions in urban contexts [5]. Bogotá can be included in these scenarios, especially when it comes to the design and evaluation of comprehensive public policies.

**Fig 1:**
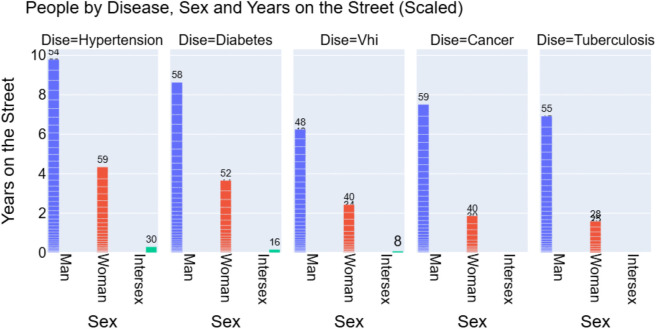
Number of people by gender, disease type, and time living on the street.

Diseases among the homeless population in Bogotá require in-depth study aimed at highlighting their high health and social vulnerability, which directly impacts mortality, community transmission, and public policies. Fig 1 shows that most individuals—especially men—suffer from chronic diseases such as hypertension and diabetes, as well as communicable diseases like HIV/AIDS, tuberculosis, and cancer, after an average of over five years living on the streets. This reflects severe deficiencies in access to healthcare, early diagnosis, and treatment continuity, as well as an intense stigmatization that deepens social exclusion. The lack of differentiated care pathways for women and intersex individuals further reinforces the existing inequalities in care. This scenario demands urgent strategies, including mobile screening, comprehensive care with a differential approach, and AI-powered tools to prioritize critical cases and optimize resources.

**Fig 2:**
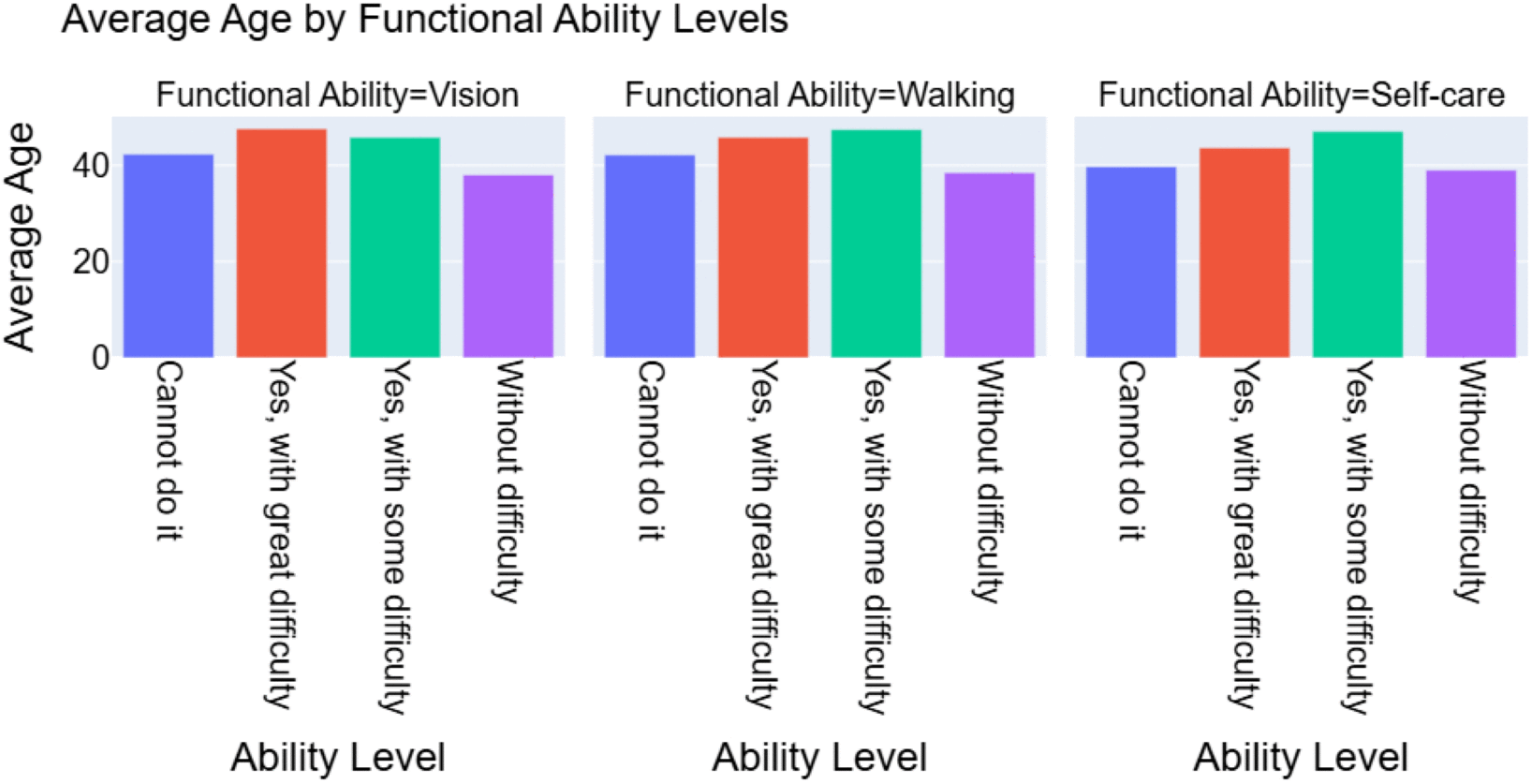
Ability to see, walk, and care for oneself according to age.

Additionally, homeless individuals exhibit varying levels of difficulty in basic functional skills such as vision, mobility, and self-care, which are associated with a progressive increase in the average age. Fig 2 shows that those who report severe difficulties (“Yes, with great difficulty”) are older on average, which reflects cumulative physical deterioration and loss of autonomy over the years on the street. These skills are essential for daily survival under adverse conditions, as they determine the ability to move, search for food, access social services, and find shelter in safe spaces. The loss of functional autonomy exacerbates vulnerability to violence, accidents, and limited access to medical care. To mitigate this issue, the State and the Mayor’s Office of Bogotá must strengthen comprehensive health programs, including mobile geriatric assessments, functional rehabilitation, the provision of assistive devices (such as canes or wheelchairs), and social reintegration initiatives with a focus on the aging population.

**Fig 3:**
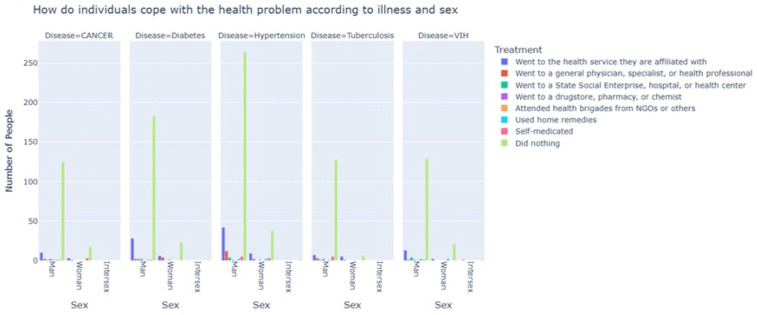
What homeless individuals do to access healthcare services.

Furthermore, Fig 3 reveals a particularly alarming trend: the most common response across all groups is “did nothing”, exposing a serious gap in healthcare coverage, adherence, and support. This trend is especially evident among men, who account for the majority of cases, followed by women and, to a lesser extent, intersex individuals. The limited use of formal healthcare services, either through a health insurance provider or public hospitals, indicates significant structural barriers, including stigma, bureaucratic procedures, and lack of information. The low engagement with mobile health brigades or private professionals suggests a deep disconnection between the studied population and the primary healthcare strategies currently in effect. This increases the risk of complications, premature death, and the community transmission of diseases such as tuberculosis and HIV/AIDS. These findings suggest substantial barriers to healthcare access and continuity of care among the study population, particularly in accessing formal healthcare services and adhering to preventive interventions. with mobile medical brigades, awareness campaigns, and active case finding while also ensuring simplified and tailored pathways for diagnosis and treatment. These efforts must be coordinated with Non-governmental organizations and community health networks to improve quality of life and public health.

**Fig 4:**
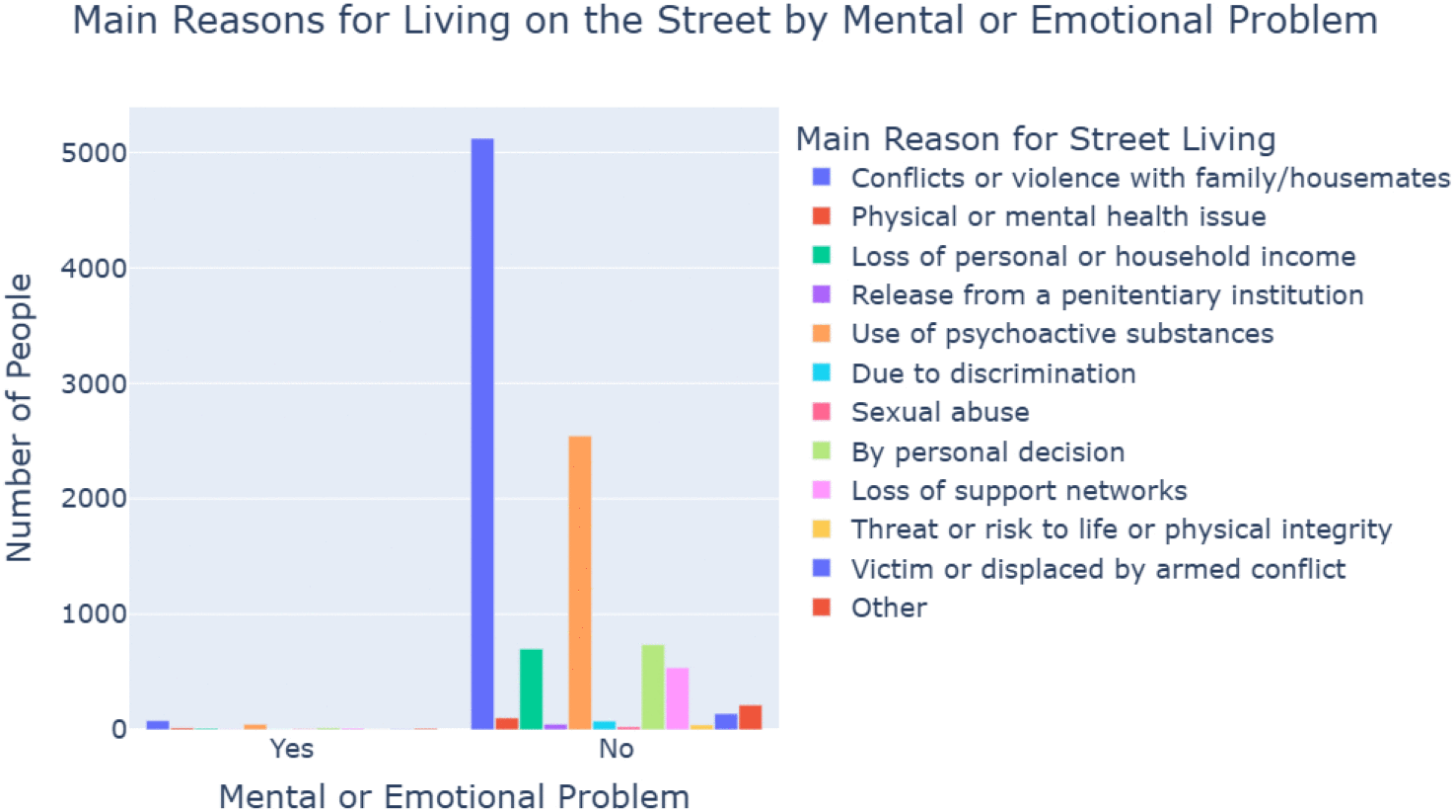
Main reasons for living on the streets.

Another important aspect for this study was the reason for living on the streets. According to Fig 4, most of the homeless people in Bogotá do not report mental or emotional problems as the main cause. Instead, family conflict or violence is the most frequent reason, with over 5000 cases, followed by income loss and the use of psychoactive substances. Among those who do report mental or emotional problems (a smaller group), domestic violence and the loss of support networks remain the key causes. The occurrence of sexual abuse and discrimination indicates extreme vulnerability. These findings underscore the need to strengthen preventive strategies focused on family protection, substance abuse prevention, and a comprehensive mental health care that offers early interventions and differentiated pathways for victims while promoting dignity and access to basic rights.

**Fig 5:**
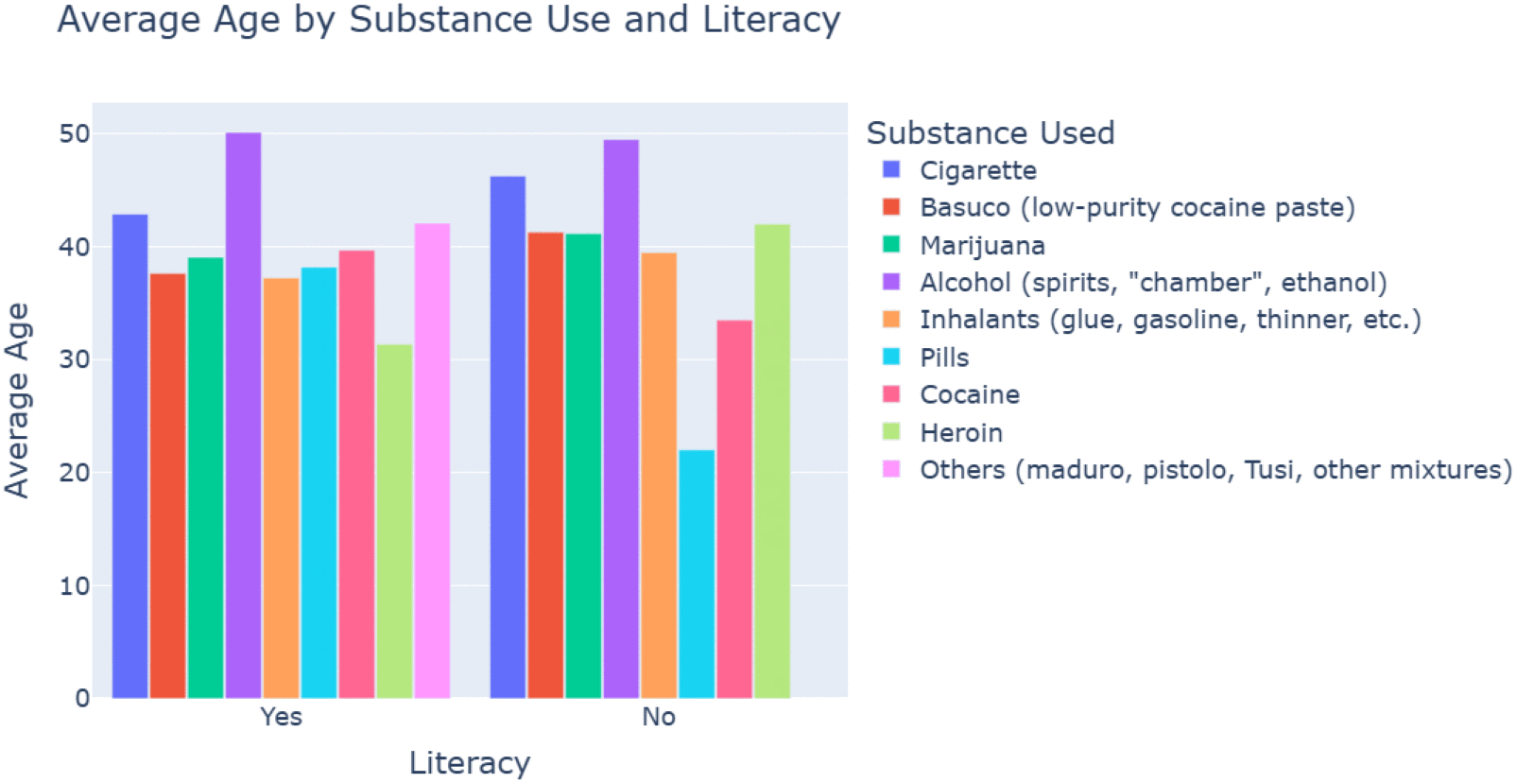
Main psychoactive substances used.

Fig 5 shows the differences in terms of psychoactive substance use by average age and literacy level among the studied homeless individuals. Those who cannot read or write tend to consume substances like cigarettes, alcohol, and heroin at older ages, likely reflecting educational exclusion and cumulative deterioration. On the other hand, pill use displays an inverse pattern, with significantly younger average ages among illiterate individuals, which suggests early vulnerability to more accessible and dangerous substances. Among the literate individuals, a higher consumption of alcohol, cigarettes, and mixtures (such as *tusi*) stands out, possibly related to prior urban socialization and exposure to substance use. These dynamics reflect a close relationship between the lack of educational opportunities, marginalization, and substance type. To address this issue, it is crucial to strengthen functional literacy programs and psychosocial support on the streets, in combination with public health policies and social reintegration strategies. Including age- and substance-specific educational and preventive components could reduce medical and social complications and improve recovery prospects for this population.

**Fig 6:**
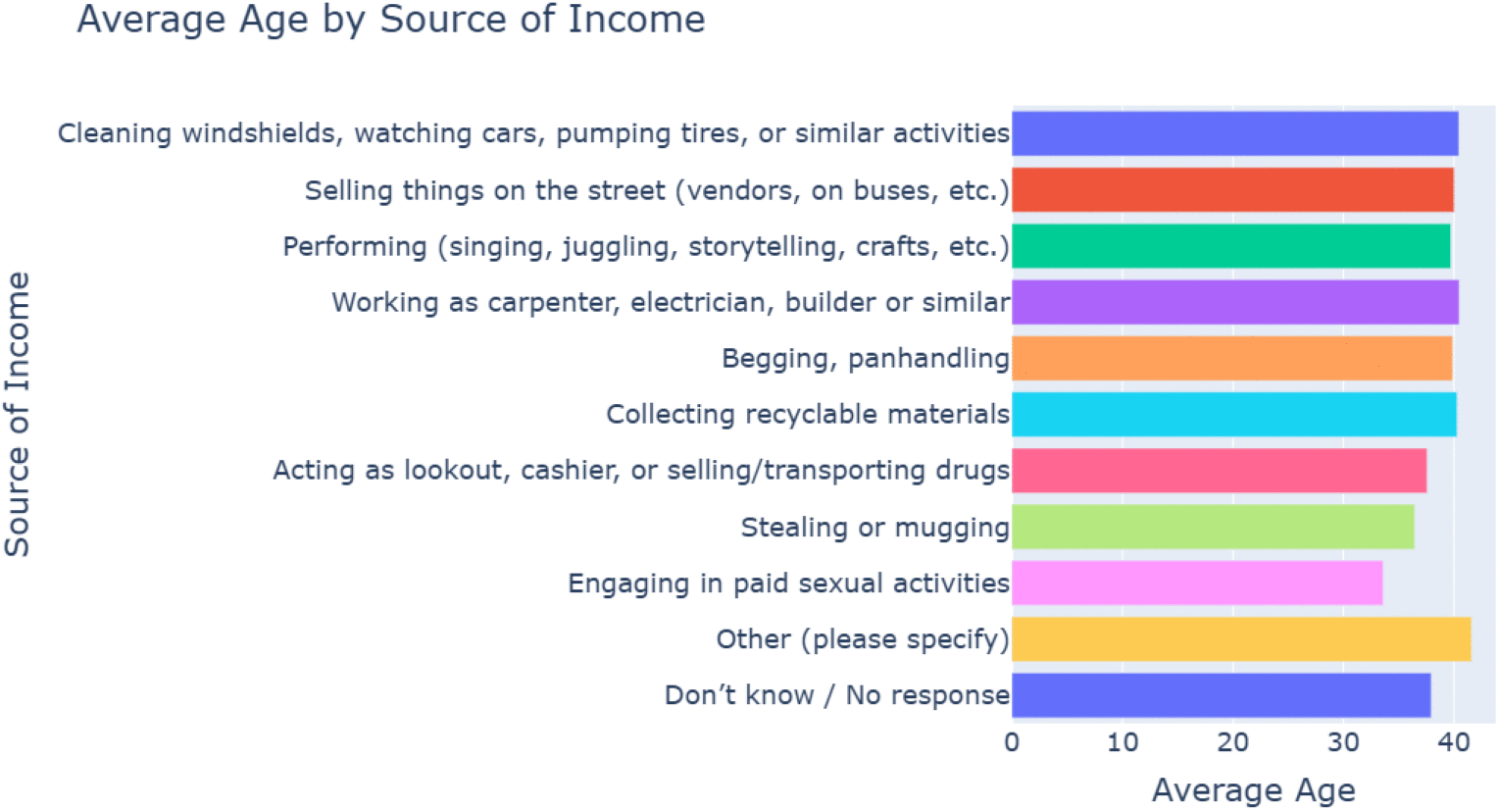
How homeless individuals earn a living.

A key factor for remaining on the streets is income. Fig 6 shows that the main sources of income among homeless individuals in Bogotá include window cleaning, street vending, artistic performances, and begging. These activities are mostly performed by adults around 40 years old, indicating a predominantly working-age population. Other activities include the collection of recyclable materials and informal work in construction or electricity, reflecting precarious labor conditions and constant efforts to survive. Conversely, high-risk or illegal activities, such as theft, drug transportation, or sex work, are associated with slightly younger average ages, suggesting that younger individuals are more likely to become involved in illicit or highly vulnerable situations. The wide distribution of activities highlights a lack of formal opportunities and social protection, forcing people into informal and dangerous jobs. This scenario underscores the need to implement comprehensive programs for labor inclusion, job training, and the generation of dignified income, in coordination with health and psychosocial support policies.

The study of homeless populations has gained increasing relevance in the academic literature due to its profound implications for public health, social exclusion, and urban planning. Several studies have explored risk factors and care needs. For instance, Fazel, Geddes, and Kushel [14] observed a high prevalence of chronic and mental illness among homeless individuals in high-income countries. Baggett *et al.* [6] highlighted a limited access to healthcare and high mortality from preventable conditions. Furthermore, Cassone [25] reported high rates of tuberculosis, hepatitis C, and HIV/AIDS in this population. In the AI context, Vajiac *et al.* [15] developed predictive models to prioritize subsidies and prevent evictions that could lead to homelessness. Similarly, Wilder *et al.* [16] applied AI to optimize HIV/AIDS prevention among the homeless youth. Rahman and Chelmis [17] implemented algorithms to identify the homeless individuals with the highest public service costs, while Kube, Das, and Fowler [18] used machine learning models to allocate personalized social services. Furthermore, Shaker *et al.* [9] analyzed shelter system trajectories using machine learning, and Esteva *et al.* [12] discussed deep learning’s capacity to support early disease detection in marginalized populations. Hong *et al.* [19] predicted shelter re-entry using AI, while Kube, Das, and Fowler [18] applied administrative models to forecast chronic homelessness. Rube, Das, and Fowler [20] also introduced resource prioritization systems for the homeless in Los Angeles, and Rahman and Chelmis [17] used predictive analysis for early interventions. Likewise, Esteva *et al.* [12] reviews the potential of AI models to monitor and predict clinical outcomes in vulnerable populations.

Although these research works offer valuable contributions, significant gaps remain. Most studies focus on high-income countries and fail to specifically address the intersection of homelessness, chronic and communicable diseases, and local sociodemographic factors. In the case of Bogotá, with its cultural and migratory diversity and its high prevalence of diseases like tuberculosis, HIV/AIDS, and cancer among the homeless, there is a lack of research integrating AI to predict health risks in a contextualized manner. This underscores the urgency and relevance of developing an AI-based disease prediction model for the homeless population of Bogotá, which could serve as a key tool for designing more accurate public policies, optimizing resource allocation, and reducing inequalities in healthcare access and reintegration programs.

## Materials and methods

This paper presents the results of an observational, analytical, and predictive study based on the secondary analysis of census data. This study followed a quantitative approach and was structured according to the CRISP-DM methodology, as outlined below:

- **Context and setting.** This research was carried out in Bogotá, Colombia, within an urban environment characterized by high social vulnerability and a great relevance of epidemiological surveillance and public health.
- **Study population.** This study included homeless individuals in the city who were registered during the 2024 Homeless Census.
- **Sampling and collection techniques.** A census sampling approach was used, analyzing all available records without probabilistic selection.
- **Sample size.** The analysis was based on 10 478 individual records and 46 demographic, clinical, functional, and socioeconomic variables.
- **Model and analytical techniques.** Multiple machine learning algorithms were implemented and compared. XGBoost was selected due to its superior balance regarding accuracy, sensitivity, and the F1-score.
- **Data analysis.** The data underwent cleaning, normalization, and encoding processes, followed by a 70/30 split for training and testing, a cross-validation for hyperparameter tuning, a performance evaluation using standard metrics, and an interpretability analysis using SHAP values.

**Fig 7:**
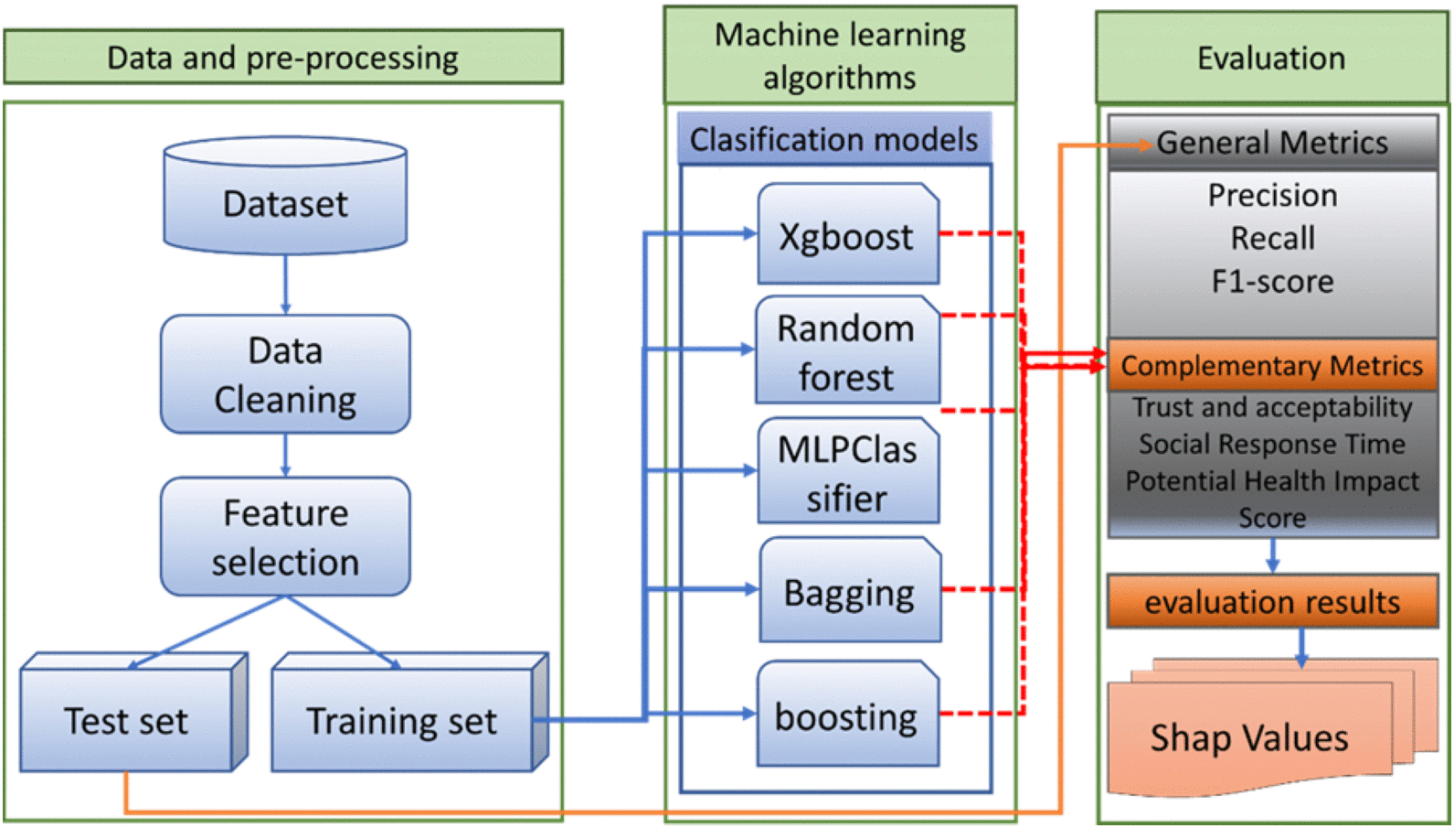
Proposed model.

The proposed model is based on Ai in the form of machine learning techniques. Five classification algorithms were implemented: XGBClassifier, RandomForestClassifier, MLPClassifier, BaggingClassifier, and AdaBoostClassifier, which are specifically tailored for classification problems (Fig 7). Each model component is described below.

- **Data acquisition.** This component handles the end-to-end management of data from acquisition via the open data platform of the National Administrative Department of Statistics (DANE) to pre-processing. The data consist of publicly available information in formats that allow for free use and reuse under open licenses and without legal restrictions. This process facilitates the creation of a dataset that contains all necessary variables for training and deploying the model.
- **Dataset.** The data come from the 2024 Bogotá Homeless Census, a statistical tool led by the District Secretariat for Social Integration with support from agencies such as DANE and IDIPRON, in an attempt to characterize the homeless population and guide evidence-based public policy [26].

The target variable (“Class”) was defined as a multi-class categorical variable representing the disease status reported in the census. Six mutually exclusive classes were established: (0) no reported disease, (1) hypertension, (2) diabetes, (3) HIV/AIDS, (4) tuberculosis, and (5) cancer.

The original dataset allowed an individual to report multiple health conditions (comorbidities). In order to ensure the consistency of the multiclass classification approach and avoid overlap between categories, a clinical hierarchy strategy was implemented based on criteria of epidemiological severity and public health relevance. In cases where a person had more than one disease, the class corresponding to the greatest clinical severity was assigned, prioritizing high-impact communicable diseases (HIV/AIDS and tuberculosis), followed by cancer and, subsequently, chronic noncommunicable diseases such as hypertension and diabetes.

This procedure allowed for the construction of mutually exclusive classes, ensuring methodological consistency during model training and preserving analytical relevance from a public health perspective.

In addition, a comprehensive data dictionary was developed documenting the 46 variables included in the analysis. For each variable, the following were specified: name, conceptual description, data type (numeric or categorical), coding scheme, and transformations applied during the preprocessing stage. The variables were organized into five main domains:

- Sociodemographic characteristics: age, sex, type of document, and municipality of origin.
- Functional abilities: ability to see, walk, perform self-care activities, and literacy level.
- Socioeconomic conditions: sources of income, work activities, and time spent living on the streets.
- Physical and mental health conditions.
- Behavioral factors: including psychoactive substance use.
- To minimize the risk of information bias and predictor-outcome circularity, all variables directly used to construct the target variable (“Class”) were excluded from the predictive feature set during model training. Specifically, self-reported diagnoses corresponding to the final assigned disease category (e.g., HIV/AIDS, tuberculosis, cancer, hypertension, or diabetes) were not simultaneously used as independent predictors for the same classification outcome.
- The outcome variable was defined as the dominant disease risk category assigned according to a predefined epidemiological hierarchy derived from census responses, rather than the binary presence or absence of a single condition. In contrast, predictor variables comprised exclusively sociodemographic, behavioral, functional, and contextual characteristics that did not directly define the target class.
- Health-related predictor variables referred exclusively to secondary contextual conditions or functional health indicators excluded from target class assignment. This procedure was implemented to reduce the possibility of data leakage, artificial inflation of predictive performance, and circular associations between predictors and outcomes.

- **Data cleaning.** This step is carried out using the CRISP-DM methodology [27], beginning with an exploratory data analysis to understand the dataset. Irrelevant columns and duplicate records are removed. Missing values are imputed using the average of the preceding and following entries. Records that still contain null values are discarded. For classification purposes, normalization is applied using MinMaxScaler, scaling the values to a range between 0 and 1. Subsequently, the features are transformed through categorical variable encoding and one-hot encoding, enabling the binary representation of categorical data. This step is crucial for improving the distribution of categorical information and preparing the data in a format that is more suitable for the model.
- **Feature selection.** This step is essential for transforming raw information into more relevant and useful variables for analysis. From scattered or unstructured data, new variables are generated to better reflect the population’s real conditions—such as time spent on the street, the types of support received, or substance use history. This process helps to interpret complex data by converting them into numerical or categorical formats, reduces noise and redundancy by eliminating irrelevant columns, and enables the integration of institutional sources through the standardization of key variables.
- **Training set (70%).** This set is used to train the models and learn patterns, applying cross-validation and hyperparameter tuning without exposure to unseen data.
- **Test set (30%).** This dataset is used to evaluate the final performance of the model with previously unseen data, thereby ensuring objective validation.

Table 1 presents the dataset variables, which characterize the homeless population of Bogotá based on multiple dimensions. These include demographic data (age, gender, ID type, usual residence), functional abilities (seeing, walking, self-care, literacy), education level, and income generation methods. They also cover the street life trajectory (years, municipality of origin, reasons for entering and remaining on the streets), access to aid (food, shelter, healthcare, emotional and psychosocial support), family ties, and both physical and mental health conditions (diseases, suicide attempts). Additionally, substance use patterns, the frequency and type of consumption, daily survival strategies, job skills, and employment history are recorded. Altogether, these variables provide a comprehensive view of the context and needs of the homeless population.

**Table 1:** Dataset variables.

Variable	Description
Where have you lived most of the time	Usual place of residence (street, house, shelter, etc.)
ID type	Type of identification (ID card, minor card, no document)
Gender	Gender identification (male, female, intersex)
Age	Age in completed years at the time of the census
Can see	Visual capacity
Can walk	Motor ability for movement
Can use hands	Fine motor skill
Can take care of self	Level of autonomy
Can relate to others	Ability to socialize or interact
Can read and write	Basic literacy level
Education level	Highest level reached in the education system
How do you earn money	Income source (informal work, aid, recycling, etc.)
Years living on the street	Time since entering homelessness
Municipality where street life began	Place where homelessness started
Main reason for living on the street	Initial reason (family conflict, addiction, unemployment, etc.)
Reason for continuing to live on the street	Current reason (addiction, lack of opportunities, etc.)
Receives financial aid	Whether financial support is received
Receives food aid	Access to food through support
Receives shelter aid	Access to shelter or refuge
Receives psychosocial support	Emotional and social assistance
Receives addiction treatment	Access to substance abuse treatment
Receives emotional/affective support	Support related to relationships and affection
Receives medical care	Access to health services
Receives hygiene support	Access to personal hygiene services
Contact with family of origin	Maintains relationship with family members
Mental/emotional problems, suicide attempt	Diagnosed or reported emotional conditions
Sought medical care	Access or attempts to seek treatment
Diabetes	Presence of diabetes
Hypertension	Presence of hypertension
Cancer	Presence of cancer
Tuberculosis	Presence of tuberculosis
AIDS	Presence of HIV/AIDS
Smokes cigarettes	Indicates cigarette use
Uses “basuco”	Indicates use of “basuco” (cocaine residue)
Uses marijuana	Indicates marijuana use
Drinks alcohol	Indicates alcohol use
Uses inhalants	Indicates inhalant use
Takes pills	Indicates pill use
Uses cocaine	Indicates cocaine use
Uses heroin	Indicates heroin use
Main substance used	Primary substance of use
Frequency of basuco use	Frequency of specific substance use
How food is obtained	Means of accessing daily food
Skills and abilities	Tasks or activities they can perform (recycling, art, etc.)
Work history	Whether they have had formal or informal employment
Disease	Presence of any of the above-mentioned illnesses

Table 2 describes each of the machine learning algorithms implemented in the model.

**Table 2:** Machine learning algorithms implemented in the model.

Algorithm	Description
XGBoost	Based on ensemble learning, XGBoost integrates multiple models to achieve higher prediction accuracy. Errors made by earlier models are corrected by adjusting subsequent models through weighted contributions. This approach ensures effective model improvement through iterative boosting [32].
Random Forest	Also based on ensemble learning, this algorithm builds multiple decision trees trained independently and in parallel. Each tree is constructed using a random sample of the dataset and a random subset of features at each split, introducing diversity among trees. Final predictions are made via majority voting (for classification), which reduces variance and improves the overall model’s stability and accuracy [33].
MLPClassifier	Based on multi-layer perceptron (MLP) neural networks. It uses supervised learning to map inputs to outputs by learning nonlinear functions through one or more hidden layers. Each neuron performs a linear combination of inputs followed by an activation function. Weights are adjusted through backpropagation by minimizing cross-entropy loss. This model supports multi-class and multi-label classification. It requires feature scaling and hyperparameter tuning (such as the number of layers, neurons, and learning rate). Final outputs are obtained through implicit voting via softmax or sigmoid functions.
Bagging	Bagging (Bootstrap Aggregating) improves predictive performance compared to a single model by creating multiple instances of a base classifier (e.g., decision trees or linear regression). The goal is to learn from a group of predictors (experts) and combine their votes. It reduces the variance of predictions by averaging over multiple diverse models. Bagging is a homogeneous ensemble of weak learners trained independently in parallel and combined to produce the final prediction via averaging (for regression) or voting (for classification) [34].

The model, datasets, and notebooks used in the development of this research can be found at the following link: https://github.com/hugo77/Calle_2025_Enfermedades. The dataset is anonymized and made publicly available by the Bogotá Mayor’s Office in various formats for use and consultation (“The exposed data is public information provided in formats that allow its use and reuse under an open license and without legal restrictions for its utilization. The responsibilities for its use are established in Law 1712 of 2014, the Law on Transparency and the Right of Access to National Public Information”) [23].

## Results

The evaluation of our model was divided into two parts: the selection of the best-performing machine learning algorithm based on standard classification metrics such as precision, recall, and the F1-score (as summarized in Table 3), and the assessment of the selected algorithm using complementary evaluation metrics, which are described in Table 4.

**Table 3:** Classification metrics.

Metric	Equation	Description
Precision	$\text{Precision}=\;\frac{TP}{TP+FP}$	Measures how many of the instances classified as positive are actually positive
Recall	$\text{Recall}=\;\frac{TP}{TP+FN}$	Measures how many of the actual positive instances were correctly identified.
F1-Score	$\text{F1}-\text{Score}=\;\frac{Precision\times Recall}{Precision+Recall}$	The harmonic mean between Precision and Recall, balancing both values.

**Table 4:** Complementary evaluation metrics.

Metric	Equation	Description
TAS (Trust and Acceptance Score)	$\frac{\text{1}}{n}\sum_{i=\text{1}}^{n}S_{i}$	Average score of confidence and willingness to accept the model, evaluated among vulnerable populations and social stakeholders [35].
SRT (Social Response Time)	$\frac{\text{1}}{m}\sum_{j=\text{1}}^{m}\left(T_{ij}-\;T_{aj}\right)$	Average time between the alert issued by the model and the effective social intervention [36].
PHIS (Potential Health Impact Score)	$w_{\text{1}\;}.\;R_{eventos}+w_{\text{2}}\;.\;A_{vida}+\;w_{\text{3}}\;.\;A_{aderencia}$	Weighted index measuring the potential benefit to general health and quality of life derived from early actions [37].

### Selection of the best-performing algorithm

In this process, the metrics described in Table 3 were used. To ensure a fair comparison, hyperparameter tuning was performed for all algorithms using the *GridSearchCV* function from Pyhton’s *scikit-learn* library, as shown in Table 4. This table shows that the XGBoost model achieved the best results across all evaluation metrics.

A comparative analysis of machine learning algorithms (Table 5) showed that, although Bagging and AdaBoost achieved slightly higher accuracy values, XGBoost demonstrated the most balanced overall performance when considering precision, recall, and F1-score combined, outperforming Random Forest, Bagging, AdaBoost, and MLPClassifier on this criterion. This balance was considered particularly relevant in the context of public health surveillance, where minimizing false negatives is critical for identifying individuals requiring priority intervention.

**Table 5:** Algorithm results based on evaluation metrics.

Algorithm	Best Hyperparameters	Precision	Recall	F1-Score
XGBoost	{‘learning_rate’: 0.05, ‘max_depth’: 3, ‘n_estimators’: 100, ‘subsample’: 0.8}	0.91	0.57	0.70
Random Forest	{‘bootstrap’: False, ‘max_depth’: None, ‘max_features’: ‘sqrt’, ‘n_estimators’: 100}	0.91	0.55	0.68
MLPClassifier	{‘activation’: ‘relu’, ‘alpha’: 0.001, ‘hidden_layer_sizes’: (100,), ‘learning_rate’: ‘constant’, ‘solver’: ‘adam’}	0.61	0.40	0.66
Bagging	{‘bootstrap’: False, ‘max_features’: 0.5, ‘max_samples’: 0.8, ‘n_estimators’: 100}	0.98	0.54	0.66
AdaBoost	{‘estimator__max_depth’: 3, ‘learning_rate’: 0.5, ‘n_estimators’: 50}	0.98	0.55	0.67

Although Bagging and AdaBoost achieved higher precision scores (0.98), they did so at the cost of substantially lower recall (0.54 and 0.55, respectively), indicating a greater number of false negatives — cases in which the model fails to identify individuals with a disease. This is especially problematic in public health contexts, where missed diagnoses may carry serious or irreversible consequences. XGBoost, in contrast, obtained the highest F1-score (0.70), reflecting the most favorable balance between precision (0.91) and recall (0.57) among the evaluated models.

Although the model achieved moderate sensitivity (0.57), this result should be interpreted cautiously given the highly heterogeneous and socially vulnerable nature of the study population. Rather than representing optimal clinical screening performance, XGBoost should be understood as a complementary decision-support tool capable of assisting prioritization processes and epidemiological surveillance. Future studies should compare its predictive capacity against traditional screening and outreach strategies currently used in homeless healthcare programs.

From a public policy perspective, XGBoost’s balanced predictive capacity enables the timely and accurate identification of individuals who require priority healthcare, facilitating early intervention. This feature is critical in vulnerable populations such as people experiencing homelessness, where harsh living conditions can accelerate health deterioration and limit access to medical services. A model like XGBoost can be integrated into epidemiological surveillance systems or social care platforms, allowing health and governmental authorities to anticipate outbreaks, allocate resources more efficiently, and design targeted prevention and treatment strategies.

Furthermore, XGBoost’s robustness in handling noisy or incomplete data, which are common in records of the homeless population, makes it especially suitable for real-world scenarios. For these reasons, XGBoost not only demonstrates superior performance in traditional metrics, but also a high practical applicability in the design and implementation of public health policies focused on equity, health, and social inclusion.

### Evaluation using complementary metrics

These findings indicate that technical predictive performance alone is insufficient to guarantee effective implementation in real-world public health settings. Instead, the results highlight the need for complementary mechanisms such as community mediation, transparent communication, participatory implementation strategies, and stronger integration with healthcare services.

The results for XGBoost, presented in Table 6, reflect an acceptable technical performance but reveal notable social limitations. The trust and acceptance score (TAS) of 0.49 indicates a moderate-to-low perception, primarily because, despite its accuracy, the model is often perceived as a ‘black box’, *i.e.*, it is difficult to interpret by vulnerable populations and social workers, which reduces their willingness to follow its recommendations. The System Response Time (SRT) of 24.86 hours should be interpreted within the actual operational context of interventions targeting homeless populations. In traditional screening and referral schemes used by mobile brigades and district programs, the identification of a case, its validation, and coordination with health services often take several days, depending on institutional availability, intersectoral coordination, and the geographical location of the individual.

**Table 6:** XGBoost results using complementary metrics.

Metric	Value
TAS (Trust and Acceptance Score)	0.49
SRT (Social Response Time)	24.86 hours
PHIS (Potential Health Impact Score)	0.24

In contrast, the artificial intelligence-based model allows prioritization alerts to be generated approximately 24 hours after information processing, representing a substantial reduction in the time required to identify high-risk cases. In a population characterized by high mobility and discontinuity in contact with formal services, this reduction in response time can be decisive in preventing the loss of clinical follow-up and improving the opportunity for intervention.

However, although the model significantly optimizes the detection and prioritization phase, its ultimate impact continues to depend on external structural factors, particularly the logistical capacity of the health system and the levels of institutional trust that condition the effective implementation of interventions.

These results demonstrate that, while XGBoost is technically robust, its real-world effectiveness in social contexts depends heavily on complementary strategies such as model interpretability, community mediation, and trust-building mechanisms. Without these, it is unlikely that the model will achieve a tangible and sustainable impact on vulnerable populations.

Beyond technical performance metrics, the complementary indicators revealed important operational and social limitations. The Trust and Acceptability Score (TAS = 0.49) suggests moderate-to-low levels of perceived trustworthiness and social acceptability, indicating that model predictions may encounter skepticism among vulnerable populations and frontline social workers. Similarly, the Potential Health Impact Score (PHIS = 0.24) suggests a limited operational impact under current institutional and logistical conditions.

These complementary indicators should be interpreted as exploratory operational metrics rather than standardized clinical validation measures. They were included to provide a broader perspective on the potential social and institutional challenges associated with implementing XAI-based tools in vulnerable populations.

### SHAP values

SHAP (SHapley Additive exPlanations) values, an explainability approach based on cooperative game theory, were used to interpret the XGBoost model. Specifically, SHAP is based on Shapley values, which provide a rigorous mathematical framework for fairly and consistently assigning the contribution of each variable to an individual model prediction.

In this study, the TreeExplainer method was used, optimized for models based on decision trees and ensemble algorithms such as XGBoost. SHAP values were calculated on the test set, allowing for both a global interpretation—by estimating the average importance of the variables in the set of predictions—and a local interpretation, aimed at explaining individual predictions in detail.

A fundamental property of SHAP is local additivity, which guarantees that the sum of the individual contributions of each feature exactly equals the final output of the model. Likewise, the method satisfies theoretical properties of consistency and absence of contribution for non-informative variables, strengthening the transparency, interpretability, and ethical legitimacy of the model, particularly in contexts of high social vulnerability where explainability is a key requirement for data-driven decision-making.

To open the black box of XGBoost, SHAP values were employed, as they provide a quantitative explanation for each feature’s contribution to individual predictions. This is particularly critical in sensitive domains such as the health of homeless populations, where distrust in automated systems tends to be high. SHAP help to make the prediction process transparent, highlight key risk factors, detect potential biases, and enhance social acceptance, turning a complex model into an explainable and trustworthy tool that can have a positive impact on health and quality of life.

**Fig 8:**
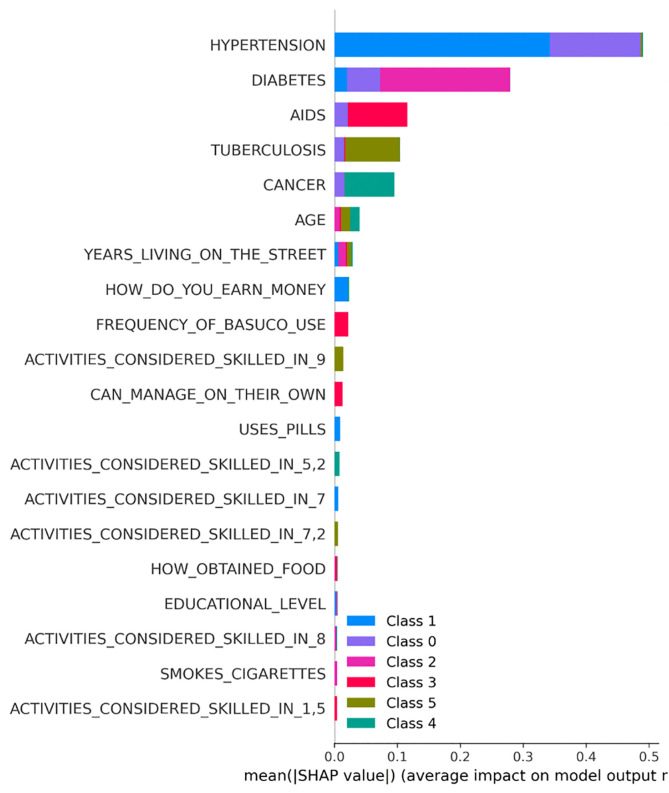
Summary plot.

Fig 8 shows how various features in the dataset contribute to the predictive model, revealing that chronic diseases have the highest impact on classification outcomes. Variables such as hypertension and diabetes are especially prominent in classes 1 and 2, which underscores their importance in identifying high-risk profiles and critical health conditions. The presence of HIV/AIDS and tuberculosis also emerges as a key determinant, often associated with classes that likely represent severe or infectious clinical states, while cancer appears as an indicator of serious illness across several outcome classes.

Although of lower relative importance, age contributes to differentiating levels of vulnerability, particularly among older individuals. Behavioral and socioeconomic factors—such as the time spent living on the streets; the use of substances like basuco, cigarettes, or pills; and sources of income—complement the model’s explanatory power, reflecting patterns of accumulated risk and functional deterioration.

Lastly, variables related to functional abilities (such as skills or qualified activities) and educational level show lower contribution scores, suggesting less discriminative power in this context.

Taken together, the interpretation provided by SHAP values demonstrates that clinical health status is the primary differentiator in predictions for the homeless population, while social and functional dimensions help to refine said predictions. This layered understanding supports the targeted design of interventions and the development of evidence-based public policies aimed at improving health outcomes for this vulnerable group.These results are directly connected to social impact metrics such as the TAS. An explainable model grounded in clear clinical variables can significantly improve professionals’ trust and community acceptance by providing transparency in risk prioritization. Regarding the SRT, the early identification of critical factors through SHAP enables a faster prioritization of interventions, optimizing resources for individuals with the most urgent conditions—such as uncontrolled hypertension or HIV/AIDS. Lastly, the PHIS reflects the model’s capacity to direct preventive and therapeutic actions towards subgroups with a higher probability of severe health deterioration, thereby strengthening public policy and differentiated care programs aimed at reducing morbidity and mortality among the homeless population.

**Fig 9:**
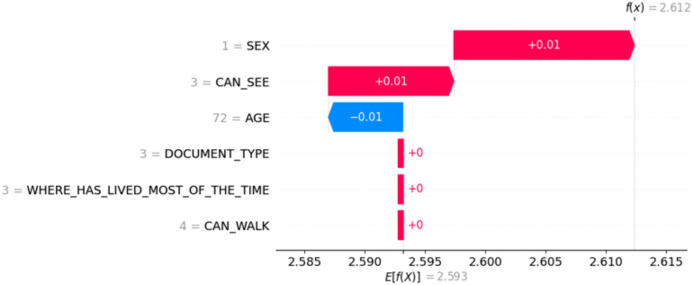
SHAP waterfall plot.

Together, the combination of model outputs with these social and health metrics enhances the effectiveness and legitimacy of intervention strategies. The SHAP waterfall plot in Fig 9 illustrates how each individual feature of record 20 contributes to the model’s final prediction, starting from the base expected value E[f(x)] = 2.593 and reaching the final output F(x) = 2.612.

In this specific case, the feature SEX = 1 (likely male) and CAN_SEE = 3 (specific visual condition) each provide a positive contribution (+0.01), pushing the prediction upward. This suggests that being male and having this visual status slightly increase the likelihood of belonging to a higher-risk class or needing priority intervention. In contrast, AGE = 72 contributes negatively (−0.01), slightly reducing the final prediction score. This might indicate that, for this particular individual, older age slightly moderates the expected risk according to the model’s learned pattern. Other variables, such as DOCUMENT_TYPE, WHERE_HAS_LIVED_MOST_OF_THE_TIME, and CAN_WALK, report near-zero contributions, implying minimal influence on this individual prediction. This detailed analysis allows for a transparent interpretation of why a specific classification was made, reinforcing social trust and acceptability (TAS) by offering clear and personalized explanations. Moreover, it contributes to a SRT by enabling the precise prioritization of micro-level interventions, and it enhances the potential health impact (PHIS) by showing how individual characteristics are translated into risk-based decisions, guiding more accurate and effective policies and care strategies.

The validity and reliability of the model used in this study are supported by a systematic and transparent methodological design that aligns with good practices in data science. The analysis was based on an official data source with broad population coverage, *i.e.*, the 2024 Bogotá Homeless Census, which strengthens the external validity of the results by reducing selection bias and enabling inferences in a highly vulnerable population.

The model was developed following the CRISP-DM methodology, with explicit processes for data cleaning, variable selection, and independent data partitioning into training and test sets (70/30), thereby contributing to the reproducibility of the analysis. Model performance reliability was assessed using standard and complementary metrics, including accuracy, sensitivity, and the F1-score, prioritizing an appropriate balance between the detection of TPs and the control of FPs, a criterion of particular relevance in public health contexts. Furthermore, a systematic comparison with multiple algorithms and hyperparameter tuning through cross-validation reinforced the robustness of the selected model, and the use of interpretability techniques such as SHAP provided additional evidence of internal validity by demonstrating consistency between the model’s predictions and known epidemiological factors, strengthening confidence in its applicability and reliability to support public health decision-making.

## Discussion

The application of machine learning techniques in public health has shown considerable promise for epidemiological surveillance and risk stratification in vulnerable populations, supporting proactive decision-making based on complex data patterns [1]. In this context, the use of ensemble algorithms such as XGBoost, in combination with interpretability mechanisms (*e.g.*, SHAP), represents a methodological trend in explainable predictive models, as observed in studies addressing both socioeconomic determinants and clinical outcome prediction [2,3]. Likewise, recent literature on equity in AI implementation emphasizes that transparency and bias assessment are essential to prevent the perpetuation of existing inequalities [4], underscoring the relevance of the ethical analysis included in this work.

Although the evaluated model demonstrated a reasonable balance between precision and recall, its predictive performance should not be interpreted as equivalent to that of a clinical diagnostic tool. In particular, the observed sensitivity of 0.57 indicates that a substantial proportion of true positive cases may remain undetected. Consequently, the system is better understood as a complementary prioritization and surveillance mechanism intended to support, rather than replace, existing screening and outreach practices within public health and social intervention programs.

Compared to research works applying similar methods in other health domains such as hospital mortality prediction, where predictor contributions are explained using SHAP to enhance clinical trust, the model’s results demonstrate a reasonable balance between accuracy and sensitivity [5], albeit with some limitations in social and trust-related metrics that reflect common challenges in applying AI to populations with heterogeneous and non-clinically standardized data. Consistent with reviews on AI and equity, the integration of additional metrics to assess social impact and ethical outcomes constitutes a significant contribution of the study, as it highlights the need to go beyond traditional technical performance metrics [4].

Moreover, broader investigations examining the impact of AI-based predictive models on health systems indicate that these approaches can improve resource allocation and tailor intervention policies when combined with participatory processes and continuous epidemiological surveillance [6]. Similarly, this article highlights the potential of the proposed model to support the prioritization of public health interventions targeting homeless populations, despite the fact that the lack of external validation and the reliance on cross-sectional census data limit the direct generalizability of the findings.

Finally, studies on bias and equity in public health models indicate that, in the absence of clear bias mitigation strategies and subgroup-level evaluations, predictive models may reproduce—or even exacerbate—existing disparities [7]. Although this article incorporates explainable analyses that identify clinical and social risk factors, the evidence suggests that future iterations of the model should include more comprehensive equity analyses and validations across different contexts in order to strengthen its overall applicability and impact.

An additional relevant limitation of this study relates to the census-based and self-reported nature of the data used. Information regarding health conditions, psychoactive substance use, history of HIV/AIDS, tuberculosis, and mental disorders was reported directly by respondents, which may introduce systematic biases associated with social stigma, fear of discrimination, and mistrust of institutions.

In homeless populations, certain highly stigmatized conditions—such as HIV/AIDS, problematic substance use, or suicide attempts—are likely to be underreported due to social desirability phenomena or fear of possible legal and social repercussions. This underreporting can lead to an incomplete representation of the actual epidemiological profile, affecting both the quality of the data and the predictive performance of the model.

From a methodological perspective, artificial intelligence models trained with biased data can reproduce or even amplify pre-existing structural inequalities. When certain conditions are underrepresented in the training set, the model may under identify people at real risk or produce classifications that do not adequately reflect their health status.

Additionally, there is a risk that the risk labels generated by the model will be interpreted in a reductionist or stigmatizing manner if they are not accompanied by adequate community mediation and professional contextualization processes. In the absence of participatory strategies and psychosocial support, predictive systems could unintentionally contribute to reinforcing negative stereotypes associated with the homeless population.

Therefore, the implementation of artificial intelligence tools in this context must be accompanied by ethical oversight mechanisms, training for institutional actors, and active community participation. The community mediation approach proposed in this study is not an accessory component, but rather a fundamental condition for mitigating potential algorithmic harm and ensuring that technology functions as an instrument of social inclusion rather than exclusion.

### Implications for Precision Prevention and Public Health Prioritization

The proposed model may contribute to precision prevention strategies by helping identify subgroups at elevated risk of adverse health outcomes within homeless populations. As noted, the model functions as a prioritization tool rather than a diagnostic system, and could assist public health and social intervention teams in directing outreach efforts, mobile screening activities, and follow-up programs under conditions of limited institutional resources.

For example, individuals experiencing prolonged street exposure, functional deterioration, barriers to healthcare access, and patterns of psychoactive substance use could be prioritized for tuberculosis screening, HIV testing, chronic disease monitoring, or psychosocial interventions. Similarly, the integration of explainable predictions through SHAP values may facilitate communication among healthcare professionals, social workers, and community health workers when jointly establishing intervention priorities.

Nevertheless, these potential applications should be interpreted cautiously, since the model has not yet undergone external validation or prospective evaluation in operational environments. Consequently, the findings should be understood as preliminary evidence of the potential utility of XAI-based models for supporting context-sensitive prevention strategies in homeless populations.

## Conclusions

This study comprehensively demonstrates the feasibility and potential of AI models—particularly XGBoost—to support early disease detection among the homeless population in Bogotá. The implementation of a multiclass classification model that integrates clinical, demographic, functional, and socioeconomic variables represents a significant contribution to public health and social planning in complex urban settings. The results indicate that the model achieves a moderate and operationally useful balance between precision and recall (F1-score = 0.70). This is especially relevant when dealing with vulnerable populations, where missing critical cases can have irreversible consequences.

However, despite its promising technical results, the model still faces important social and operational challenges. The low TAS (0.49) reflects a perception of distrust towards automated systems, particularly among populations that have historically been marginalized and stigmatized. In addition, the SRT (24.86 hours) and the PHIS (0.24) indicate that technical capacity alone is not sufficient to ensure meaningful improvements in health outcomes. It is essential for predictive results to be embedded within rapid and coordinated action strategies, accompanied by social mediation and psychosocial support, in order to maximize their effectiveness.

The use of SHAP values as an interpretability tool helped to open the ‘black box’ of the model, providing a transparent view of the most influential factors behind predictions. This not only enabled the identification of key variables such as hypertension, diabetes, HIV/AIDS, and tuberculosis, but also strengthened social trust and model acceptability (TAS), a crucial factor for the successful implementation of technological tools in public health. Furthermore, the individualized interpretation of cases allows for the design of more precise and tailored interventions, potentially improving both health outcomes and quality of life.

Additionally, this study highlights the need to holistically address the social and structural determinants that perpetuate homelessness, such as the lack of employment opportunities, broken family ties, violence, and problematic substance use. Our detailed analysis of psychosocial and behavioral variables, along with health conditions, offers a more complete landscape that allows decision-makers to prioritize targeted interventions and develop more inclusive public policies that are context-sensitive and equity-focused.

Among the main strengths of this study is the use of an official, large-scale, population-wide data source, which reinforces the external validity of the results. The systematic application of the CRISP-DM methodology, the comparison of multiple machine learning algorithms, and hyperparameter tuning through cross-validation provide methodological rigor and reproducibility. In addition, the incorporation of social and impact-related metrics, together with interpretability techniques, represents an innovative approach that goes beyond the purely technical evaluation of the model.

This study, however, has several limitations that must be considered. First, the analysis is based on cross-sectional census data, which prevents the assessment of temporal dynamics or the establishment of causal relationships. Second, the absence of external validation limits the generalizability of the results to other geographic contexts or populations. Likewise, the inherent quality issues and the heterogeneity of data on homeless populations may introduce uncontrolled biases. Finally, the social metrics indicate that a strong technical performance does not automatically translate into operational impact or social acceptance.

Our findings have relevant implications for public health and policymaking. The model can support epidemiological surveillance, the prioritization of interventions, and the efficient allocation of resources in highly vulnerable populations. From an ethical perspective, our emphasis on interpretability and transparency contributes to the responsible use of AI in sensitive contexts. Furthermore, although the proposed framework may offer a replicable methodological approach for similar urban contexts, its transferability to other populations or healthcare systems requires external validation, prospective evaluation, and contextual adaptation.

The present findings should be interpreted as context-specific evidence derived from the homeless population of Bogotá rather than as broadly generalizable conclusions applicable to all vulnerable populations. Future studies should incorporate longitudinal data and external validation to strengthen the robustness and generalizability of the model. It is also necessary to expand algorithmic equity assessments and bias analyses, alongside the integration of psychosocial and community-level variables that may improve predictive validity and strengthen estimated health impacts. From an operational standpoint, the implementation of such models should be accompanied by community mediation strategies, capacity building for social intervention teams, and clear communication mechanisms to increase trust, improve institutional responsiveness, and maximize tangible benefits for the homeless population.

### Pilot implementation framework with a human-in-the-loop approach

In order to translate predictive results into concrete operational actions, a pilot implementation framework based on a human-in-the-loop (HITL) approach is proposed, in which the artificial intelligence model acts as a decision-making support tool and not as a substitute for professional judgment. Under this scheme, the model could be integrated into a digital platform used by the District Health Secretariat or by mobile street care teams. The proposed operational flow includes the following stages:

- Data entry or update: the social worker or brigade team records or updates basic information about the individual, including sociodemographic variables, time spent on the streets, reported health conditions, and psychoactive substance use.
- Automatic generation of risk profile: the system runs the XGBoost model and produces a risk classification accompanied by an individual explanation based on SHAP values.
- Interpretive visualization: the professional accesses a simplified dashboard that presents: (i) estimated risk level, (ii) main variables that influenced the prediction, and (iii) prioritization recommendations suggested by the system.
- Contextual professional assessment: the social worker compares the prediction with their direct knowledge of the case, validating, adjusting, or discarding the proposed prioritization according to the observed social and clinical context.
- Activation of the care pathway: in cases classified as high risk, priority referral to health services, medical brigades, or specialized follow-up mechanisms is coordinated.

This approach ensures that the final decision remains under human supervision, reducing the risk of uncritical automation and allowing for the incorporation of contextual variables not captured by the model. It also promotes community mediation by enabling professionals to explain the prioritization criteria in a transparent manner, strengthening institutional trust and social acceptance of the system.

**Fig 10:**
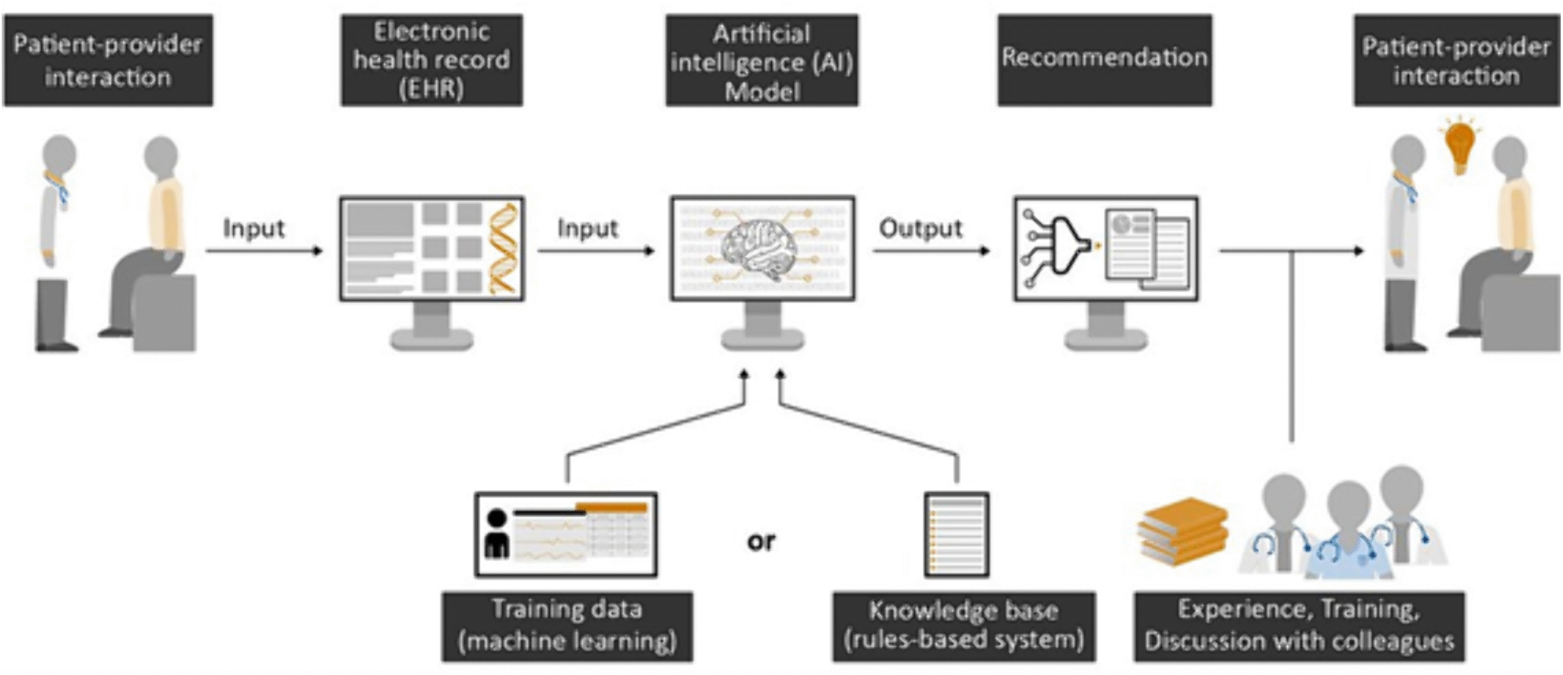
Pilot implementation framework.

The pilot implementation (Fig 10) could begin in one or two locations in Bogotá with a high concentration of homeless people, which would allow for the evaluation of operational performance, actual response times, and the level of institutional acceptance before the eventual scalability of the system.

In future works, we aim to improve the interpretability and social trust of the model by incorporating additional explainability methods such as LIME or counterfactual explanations while developing user-friendly graphical interfaces for community actors and healthcare personnel, with the goal of increasing the TAS nd the willingness to adopt the model in real-world public health settings. Future pilot implementation should include participatory evaluation with frontline social workers to assess usability, trust, and decision-making integration
