# Supplementary material for: Artificial intelligence-based prediction of diseases among homeless populations in Bogotá: Implications for targeted interventions
Source: PLoS One. 2026 Jun 26;21(6):e0352268. doi: 10.1371/journal.pone.0352268 (PMC13308786; doi:10.1371/journal.pone.0352268)
Supplement: Attachment — Submitted filename: Comments for Authors.docx [file pone.0352268.s001.docx]

**Comments for Authors**

Dear authors, you have all done a commendable job. However, the following suggestions may help strengthen the current manuscript.

1. You may write the title as “Artificial Intelligence-Based Prediction of Diseases Among Homeless Populations in Bogotá: Implications for Targeted Interventions”
2. Please write a structured abstract having sections such as Background, purpose, methods, results, conclusion, implications, and keywords.
3. Please add a short summary (what new) after abstract and before introduction.
4. Please write a clear introduction and background as separate headings.
5. Please write a clear purpose and objectives of the study.
6. Please provide information about ethics such permission for using the data.
7. Please provide information about the validity and reliability of model being used in this study.
8. Please provide references for each heading in the methodology. Provide headings such as Design, settings, population, MODEL, sampling, sample technique, sample size, analysis etc.
9. Please write a comprehensive discussion using latest papers along with compare and contrast utilizing transition words.
10. Please write conclusion, strengths, limitations, implications, and recommendations as separate headings.

Thank you
