# Supplementary material for: Artificial intelligence-based prediction of diseases among homeless populations in Bogotá: Implications for targeted interventions
Source: PLoS One. 2026 Jun 26;21(6):e0352268. doi: 10.1371/journal.pone.0352268 (PMC13308786; doi:10.1371/journal.pone.0352268)
Supplement: Attachment — Submitted filename: Response to Reviewers.docx [file pone.0352268.s004.docx]

**Dear Editors and Reviewers,**

We sincerely thank you for the time, effort, and valuable feedback provided during the review process of our manuscript. We appreciate the constructive comments and suggestions, which have significantly contributed to improving the clarity, rigor, and overall quality of the paper.

We are pleased to inform you that all the requested revisions have been carefully addressed. The main adjustments implemented in the revised version of the manuscript are summarized below (The response was highlighted using the same color applied in the article text where the corresponding change was made.):

1. You may write the title as “Artificial Intelligence-Based Prediction of Diseases Among Homeless Populations in Bogotá: Implications for Targeted Interventions”.

**The title has been revised and updated to: “Artificial Intelligence-Based Prediction of Diseases Among Homeless Populations in Bogotá: Implications for Targeted Interventions.”**

2. Please write a structured abstract having sections such as Background, purpose, methods, results, conclusion, implications, and keywords.

**The abstract has been rewritten following a structured format, including the sections: Background, Purpose, Methods, Results, Conclusion, Implications, and Keywords, to enhance clarity and compliance with the journal’s guidelines.**

3. Please add a short summary (what new) after abstract and before introduction.

**A short summary highlighting the novelty and key contributions of the study has been added immediately after the abstract and before the introduction.**

4. Please write a clear introduction and background as separate headings.

**The manuscript now includes clearly differentiated sections for the Introduction and Background, each with a well-defined focus and supporting references.**

5. Please write a clear purpose and objectives of the study.

A clear statement of the purpose and specific objectives of the study has been explicitly incorporated to guide the reader through the research scope and intentions.

6. Please provide information about ethics such permission for using the data.

Providing detailed information regarding ethical considerations and permissions related to the use of the dataset employed in the study.

7. Please provide information about the validity and reliability of model being used in this study.

Information regarding the validity and reliability of the predictive models has been expanded, including model evaluation strategies, performance metrics, and robustness considerations.

8. Please provide references for each heading in the methodology. Provide headings such as Design, settings, population, MODEL, sampling, sample technique, sample size, analysis etc.

The Methodology section has been reorganized and expanded, with clearly defined subsections such as: Design, Setting, Population, Model, Sampling, Sample Technique, Sample Size, and Analysis. Appropriate and up-to-date references have been included for each subsection.

9. Please write a comprehensive discussion using latest papers along with compare and contrast utilizing transition words.

The Discussion section has been substantially revised and expanded, incorporating recent and relevant literature. Comparative and contrasting analyses have been strengthened using appropriate transition words to improve coherence and critical interpretation of the results.

10. Please write conclusion, strengths, limitations, implications, and recommendations as separate headings.

The manuscript now presents Conclusion, Strengths, Limitations, Implications, and Recommendations as separate and clearly labeled sections, providing a comprehensive and transparent closing of the study.

We believe that these revisions have significantly strengthened the manuscript and aligned it fully with the reviewers’ recommendations. We sincerely hope that the revised version meets the journal’s standards and expectations.

The manuscript was reviewed for language and writing quality by an expert professional editor. The corresponding certificate is attached.

Thank you again for your insightful comments and for the opportunity to improve our work. We remain at your disposal for any further revisions or clarifications that may be required.

Kind regards,

Juan Sebastian Gonzalez Sanabria

On behalf of the authors
