# Supplementary material for: Artificial intelligence-based prediction of diseases among homeless populations in Bogotá: Implications for targeted interventions
Source: PLoS One. 2026 Jun 26;21(6):e0352268. doi: 10.1371/journal.pone.0352268 (PMC13308786; doi:10.1371/journal.pone.0352268)
Supplement: Attachment — Submitted filename: Reviewer matrix.docx [file pone.0352268.s003.docx]

**REVIEWER’S COMMENTS**

PLOS Global Public Health

Prediction of Diseases in Homeless People in Bogotá Using AI: Towards Intervention

| **S/N** | **Section** | **Comments from the reviewer** | **PI’s response** | **Line number** |
| --- | --- | --- | --- | --- |
| 1. | **Title** | OK | NA | NA |
| 2. | **Abstract** | Write the abstract section into strcutured sections |  | 16-30 |
|  |  | Give long form an abbreviation when its uswed for the first time |  | 16-30 |
|  |  | Please incorporate key words in your abstract |  | 16-30 |
|  |  | Should lungworm infection be included as one of the keyword? |  | 35 |
| 3. | **Introduction** | Support the introduction part with relevant data about the particular topics |  | 33-105 |
|  |  | “HVI” what does this word mean? |  | 60 |
|  |  | What was the necessity of including explanation about figures in the introduction?  Are they still part of the introduction? |  | 107-217 |
| 4. | **Materials and Methods** | Despite mentioning the five incorporate algorithm classification please show an ideal algorithm used in this study |  | 219-222 |
|  |  | Give abbreviation for the word “IDIPRON” |  | 234 |
|  |  | Did the study utilize all dataset? However its impossible as some of the data with“0” records were discarded |  | 232-235 |
| 5. | **Results** | The result part needs some wording refinement for example “achieved the best results across all evaluation metrics” technically the metrics evalution like precision can not receive best results across the evaluation process |  | 286-314 |
|  |  | Do not give interpretion section? |  | 277-397 |
|  |  | Trust and Acceptance Score often uses Likert Scale Questionaire items? What did your study applied as far as data collection techniques is concern |  | 318-324 |
| 6. | **Discussion** | Discus your results with supporting evidence from other studies. |  | 412-436 |
|  |  | Please do not include your result in the discussion section |  | 412-436 |
|  |  | Please discussion and conclusion section into different parts |  | 412-436 |
|  |  | Meanwhile discuss your results by connecting to your objectives |  | 412-436 |
| 7. | **References** | You have used some of the references which are outdated. Give references which are below 5 to 10years |  | 488-521 |
| 8. | **General comments and Questions** | What were the limitations of the study and how to mitigate them |  |  |
|  |  | Figure 1  Its not visible clearly but some numbers in the do overlap to each other.  Does the word “Vhi” a medical term or make it clear.  State the clearly the heading of Fig One |  |  |
|  |  | Please give key for Fig 1 |  |  |
|  |  | The word “Dise” stands for what? |  |  |
|  |  | Provide N for Fig 1 |  |  |
|  |  | Please revise the figures in your work they are not clearly visible, Provide N for figures, give key for each Figure, plus arrangement of the figures |  |  |
|  |  | Use consistency words such as “AIDS” has come out from nowhere in figure 8 |  |  |
|  |  | Was it necessary to include recommendations in this study? |  |  |
|  |  | Please each authors contribution in your manuscript |  |  |
|  |  | Was there any funding in this study? If yes please indicate |  |  |
|  |  | How was the confidentiality maintained in your study? |  |  |
|  |  | Please incorporates if there was any conflicting interest in your study. |  |  |
|  |  | Consider proper numbering in your manuscript |  |  |
|  |  | Please revise how to write manuscript properly as many of the important key parts were missing? For example in the authors details you provided an email contact which is not proper |  |  |
