# Supplementary material for: Artificial intelligence-based prediction of diseases among homeless populations in Bogotá: Implications for targeted interventions
Source: PLoS One. 2026 Jun 26;21(6):e0352268. doi: 10.1371/journal.pone.0352268 (PMC13308786; doi:10.1371/journal.pone.0352268)
Supplement: Attachment — Submitted filename: review comment.docx [file pone.0352268.s005.docx]

Reviewer Comments

Thanks for the opportunity to review this manuscript.

The study incorporates several social metrics (TAS, SRT, PHIS) to evaluate model performance beyond traditional technical indicators, which is innovative. It also focuses on a vulnerable population (homeless individuals) that is often overlooked, and the algorithm development is described in substantial detail.

However, several issues need to be addressed from an epidemiological and targeted prevention perspective.

First, in the Methods section, the authors should clarify how the outcome variables (e.g., HIV/AIDS, tuberculosis) and lifestyle/behavioral variables were assessed and determined, as information bias is a major concern in epidemiological studies.

Second, in the Results section, the SHAP analysis identifies hypertension, diabetes, and HIV/AIDS as "predictors," but these are also the outcome variables, raising concerns about circularity; the predictor variables and outcome variables are not clearly stated.

Third, the interpretation of the results appears overly optimistic without comparison to traditional screening methods. A recall of 0.57 is described as having a "strong ability to detect true positives," which can not convince me. The claim that XGBoost performed best across all evaluation metrics is contradicted by Table 5, where Bagging and AdaBoost achieved higher precision. The TAS (0.49) and PHIS (0.24) are repeatedly interpreted as supporting reliability and trust, but these values are low and the rationale is not provided.

Additionally, the Results and Discussion sections are not clearly separated; interpretive statements appear in both sections with similar wording. Authors should focus on objectively presenting data in the results, while interpretation should be reserved for the discussion section.

Finally, in the Discussion and Conclusions, the title emphasizes "Implications for Targeted Interventions," but concrete implications for precision prevention are not clearly articulated. The abstract statement that "Artificial intelligence models can effectively support public health through the early identification of risk profiles in vulnerable populations" is not rigorous, as the study lacks external validation and the findings are specific to the homeless population.

Besides, **since this study uses cross-sectional data, the term 'prediction' seems inappropriate. And lastly, line 404 contains a typo: 'atood out' should be corrected to 'stood out.**
