# Supplementary material for: Artificial intelligence-based prediction of diseases among homeless populations in Bogotá: Implications for targeted interventions
Source: PLoS One. 2026 Jun 26;21(6):e0352268. doi: 10.1371/journal.pone.0352268 (PMC13308786; doi:10.1371/journal.pone.0352268)
Supplement: Attachment — Submitted filename: Response to Reviewers 2.docx [file pone.0352268.s006.docx]

**Dear Editors and Reviewers,**

We sincerely thank the reviewers for their careful reading of the manuscript and for their valuable and constructive comments. Their suggestions have significantly contributed to improving the clarity, methodological rigor, and practical relevance of the study. Below, we provide a detailed, point-by-point response indicating how each observation has been addressed in the revised manuscript.

**1. Terminology and Presentation (Abstract/Highlights)**

**Reviewer Comment:** Replace “AIDS/AIDS” with “HIV/AIDS” throughout the manuscript. In the Highlights, explicitly state the value of using CRISP-DM as a structured, industry-standard methodology for data mining.

**Response:** The terminology has been revised throughout the manuscript, replacing all instances of “AIDS” with “HIV/AIDS” to ensure medical accuracy and consistency.

Additionally, the Highlights section has been updated to explicitly emphasize the adoption of the CRISP-DM framework as a structured, industry-standard methodology for data mining projects. The revised text now highlights how CRISP-DM supports methodological transparency, reproducibility, and alignment with best practices in applied artificial intelligence (**Lines 50-52**).

**2. Introduction**

**Reviewer Comment:** Sharpen the definition of the “AI gap” by noting that most prior AI research on homelessness focuses on high-income countries and explain why these models are not directly transferable to Bogotá/Latin America.

**Response:** The Introduction section has been expanded to clarify the concept of the “AI gap.” We now explicitly state that most existing AI-based homelessness research has been conducted in high-income countries such as the United States and the United Kingdom. A new paragraph explains why direct model transferability is limited, emphasizing structural differences including: healthcare system organization, socio-political and economic conditions, patterns of vulnerability and service access in Latin American contexts. This addition strengthens the contextual justification for developing locally grounded AI models (**Lines 99-117**).

**3. Methods**

**Reviewer Comment:** Provide a complete data dictionary and clearly define the target variable (“Class”). Clarify handling of comorbidities and expand the SHAP explanation, including its grounding in game theory.

**Response:** The Methods section has been substantially expanded:

A complete data dictionary has been added as a supplementary table describing variables, data types, and meanings. The target variable (“Class”) is now explicitly defined as [binary/multiclass — adjust accordingly], including a detailed explanation of label construction **(Lines 322-348)**.

The SHAP methodology description has been expanded to explain that SHAP values are derived from cooperative game theory, allowing fair attribution of each feature’s contribution to model predictions. A concise conceptual explanation and supporting references were incorporated **(Lines 457-472)**.

**4. Results**

**Reviewer Comment:** Contextualize the reported system response time (SRT) of 24.86 hours and compare it with traditional screening timelines.

**Response:** The Results section has been revised to contextualize the System Response Time (SRT) of 24.86 hours. We now compare this value with traditional public health screening processes, which typically require longer administrative and clinical coordination periods.

The revised text explains that, within interventions targeting transient and hard-to-reach populations, a response window of approximately 24 hours represents a meaningful operational improvement. This comparison highlights the practical efficiency gains enabled by the proposed AI-based approach **(Lines 435-449)**.

**5. Discussion and Limitations**

**Reviewer Comment:** Strengthen discussion of data bias from census/self-reported data and add a dedicated subsection addressing stigma and self-reporting bias.

Response: The manuscript now includes: An expanded analysis of potential biases associated with census and self-reported data. Discussion of underreporting risks related to sensitive conditions due to stigma and distrust. A new Limitations subsection specifically addressing self-reporting bias and dataset representativeness. Furthermore, we incorporated a reflection on ethical risks, noting that AI systems may inadvertently reinforce stigma if deployed without appropriate safeguards. This discussion is now explicitly connected to the proposed “community mediation” approach described in the conclusions **(Lines 582-605)**.

**6. Conclusion and Practical Implementation**

**Reviewer Comment:** Propose a pilot human-in-the-loop framework and describe how a social worker would use the model. Include a workflow diagram or mock-up.

**Response:**

The Conclusion section has been expanded to include a proposed human-in-the-loop pilot framework for real-world implementation. We now describe a practical workflow illustrating how a social worker in Bogotá could interact with the model as a decision-support tool. Additionally, a conceptual workflow diagram has been incorporated to visually represent the proposed operational process, improving the manuscript’s applied relevance and translational value **(Lines 664-694)**.

We greatly appreciate the reviewers’ insightful feedback, which has substantially strengthened the manuscript’s methodological transparency and practical applicability. All suggested revisions have been carefully incorporated, and corresponding changes have been highlighted in the revised version of the manuscript.

Sincerely,

Juan Sebastian Gonzalez Sanabria

On behalf of the authors
