# Supplementary material for: Artificial intelligence-based prediction of diseases among homeless populations in Bogotá: Implications for targeted interventions
Source: PLoS One. 2026 Jun 26;21(6):e0352268. doi: 10.1371/journal.pone.0352268 (PMC13308786; doi:10.1371/journal.pone.0352268)
Supplement: Attachment — Submitted filename: Response_Letter_26-05.docx [file pone.0352268.s007.docx]

**Dear Editor,**

We thank the editorial team for the careful and constructive review of our manuscript entitled "Artificial Intelligence-Based Prediction of Diseases Among Homeless Populations in Bogotá: Implications for Targeted Interventions." We have carefully considered all observations indicated in the manuscript and have addressed each of them below. The revised passages are presented in their updated form, along with a detailed explanation of the changes introduced.

The editorial comments corresponded to the following sections and passages, which had been highlighted in yellow in the manuscript. Each is addressed in turn.

**Comment 1 — Abstract: Results and Conclusions**

The abstract’s Results and Conclusions sections have been reviewed for clarity and precision. The phrasing “striking an adequate balance” has been revised to “achieving an operationally useful balance” to avoid evaluative ambiguity. In the Conclusions, the structure has been preserved, as it appropriately reflects both the potential and the limitations of the model. The hedging language (“may support,” “remains context-specific”) has been retained, as it is methodologically justified given the absence of external validation. No substantive content has been altered.

**Comment 2 — Background: Healthcare Access Paragraph (Fig. 3)**

We acknowledge this structural error. The passage contained an inadvertent sentence break that disrupted the logical flow. The paragraph has been revised to restore syntactic continuity. The corrected version now reads: “These findings suggest substantial barriers to healthcare access and continuity of care among the study population, particularly in accessing formal healthcare services and adhering to preventive interventions. These gaps underscore the urgent need to strengthen outreach strategies, including mobile medical brigades, awareness campaigns, and active case finding, while ensuring simplified and tailored pathways for diagnosis and treatment. These efforts must be coordinated with non-governmental organizations and community health networks to improve quality of life and public health outcomes.” The capitalization error in “Non-governmental organizations” has also been corrected.

**Comments 3 and 4 — Methods: Outcome Variable and Data Leakage Prevention**

These passages have been reformatted as continuous prose paragraphs to improve integration with the Methods section narrative. Their substantive content, which explains the epidemiological hierarchy used to construct the target variable and the exclusion criteria applied to prevent circular associations and data leakage, has been preserved in full. The revised text is as follows: “The outcome variable was defined as the dominant disease risk category assigned according to a predefined epidemiological hierarchy derived from census responses, rather than the binary presence or absence of a single condition. In contrast, predictor variables comprised exclusively sociodemographic, behavioral, functional, and contextual characteristics that did not directly define the target class. Health-related predictor variables referred exclusively to secondary contextual conditions or functional health indicators excluded from target class assignment. This procedure was implemented to reduce the possibility of data leakage, artificial inflation of predictive performance, and circular associations between predictors and outcomes.”

**Comments 5, 6, and 7 — Results: Algorithm Comparison and XGBoost Performance**

These three paragraphs have been substantially revised to eliminate redundancy, correct grammatical errors, and restore logical continuity. The incomplete sentence has been removed. The F1-score and precision-recall values are now reported only once. The article “a” before “XGBoost” has been corrected to “an” throughout. The revised version consolidates the three paragraphs as follows: “A comparative analysis of machine learning algorithms (Table 5) showed that, although Bagging and AdaBoost achieved slightly higher accuracy values, XGBoost demonstrated the most balanced overall performance when considering precision, recall, and F1-score combined, outperforming Random Forest, Bagging, AdaBoost, and MLPClassifier on this criterion. This balance was considered particularly relevant in the context of public health surveillance, where minimizing false negatives is critical for identifying individuals requiring priority intervention. Although Bagging and AdaBoost achieved higher precision scores (0.98), they did so at the cost of substantially lower recall (0.54 and 0.55, respectively), indicating a greater number of false negatives — cases in which the model fails to identify individuals with a disease. XGBoost, in contrast, obtained the highest F1-score (0.70), reflecting the most favorable balance between precision (0.91) and recall (0.57) among the evaluated models. Although the model achieved moderate sensitivity (0.57), this result should be interpreted cautiously given the highly heterogeneous and socially vulnerable nature of the study population. Rather than representing optimal clinical screening performance, XGBoost should be understood as a complementary decision-support tool capable of assisting prioritization processes and epidemiological surveillance.”

**Comments 8 and 9 — Results: Complementary Metrics**

We acknowledge that these paragraphs partially duplicated content from an earlier subsection. Upon revision, the earlier descriptive passage has been retained as the primary reporting of TAS and PHIS values, while the highlighted paragraphs have been reframed as an interpretive synthesis that contextualizes the operational and social implications of these scores. The phrase “revealed important operational and social limitations” has been introduced with the transitional marker “Beyond technical performance metrics” to signal analytical progression rather than repetition. The qualifier “exploratory operational metrics” has been preserved, as it accurately describes the non-standardized nature of these instruments.

**Comment 10 — Discussion: Clinical Interpretation of Model Performance**

This paragraph has been reviewed and refined. The phrase “the evaluated model” has replaced “the proposed model” to reflect that the model had been empirically tested by this stage of the manuscript. The expression “equivalent to that of a clinical diagnostic tool” has been retained, as it correctly frames the comparison at the level of performance standards rather than instrument type. The sensitivity value (0.57) has been explicitly cited in the text to strengthen the link between this interpretive statement and the reported results. No substantive changes to the content were required.

**Comments 11–14 — Discussion: Implications for Precision Prevention (Subsection)**

This subsection has been revised as follows. The heading has been retained with minor reformatting for consistency with the manuscript’s style. In the first paragraph, “targeted prevention strategies” has been changed to “precision prevention strategies” for terminological consistency with the heading, and the nominalization “supporting the identification of” has been simplified to “helping identify.” The caveat that the model functions as a prioritization tool rather than a diagnostic system has been introduced with the phrase “As noted” to avoid repeating the full formulation from earlier sections. In the second paragraph, “presenting” has been replaced by “experiencing” to avoid the clinical connotation of “presenting symptoms.” “Community actors” has been revised to “community health workers” for greater precision. In the third paragraph, “these applications” has been changed to “these potential applications” and “artificial intelligence” has been replaced by the abbreviation “XAI-based models” for terminological consistency with the rest of the manuscript.

**Comments 15 and 16 — Conclusions: Policy Implications and Future Directions**

These paragraphs have been lightly revised for stylistic consistency. In Comment 15, the phrase “although the proposed framework may offer a replicable methodological approach” has been retained; “additional” before “external validation” has been removed as redundant given the context. In Comment 16, the epistemological statement (“The present findings should be interpreted as context-specific evidence…”) has been repositioned to open the paragraph, so that the forward-looking recommendations follow naturally from this framing. “External validations” has been corrected to “external validation” (singular process). “Deepen equity and bias analyses” has been revised to “expand algorithmic equity assessments and bias analyses” for greater precision. “Social actors” has been replaced by “social intervention teams” for consistency with terminology used throughout the manuscript. “In order to” has been simplified to “to,” and “reduce response times” has been replaced by “improve institutional responsiveness” to align with the operational framing of the discussion.

We believe the revised manuscript is substantially improved in terms of clarity, internal consistency, and terminological precision. All changes introduced in response to the highlighted passages are reflected in the revised version of the manuscript submitted alongside this letter. We remain available to address any further questions or concerns the editorial team may have.

Sincerely,

**Juan-Sebastian Gonzalez-Sanabria**

Corresponding author: juansebastian.gonzalez@uptc.edu.co
