# Supplementary material for: Artificial intelligence-based prediction of diseases among homeless populations in Bogotá: Implications for targeted interventions
Source: PLoS One. 2026 Jun 26;21(6):e0352268. doi: 10.1371/journal.pone.0352268 (PMC13308786; doi:10.1371/journal.pone.0352268)
Supplement: Peer review history

# Peer review: Decision Letter 0

**DOI:** 10.1371/journal.pone.0352268.r001

**Authors:** Oriana Rivera-Lozada de Bonilla

16 Dec 2025

PONE-D-25-50529

Prediction of Diseases in Homeless People in Bogotá Using AI: Towards Intervention

PLOS One

Dear Dr. González-Sanabria,

Thank you for submitting your manuscript to PLOS ONE. After careful consideration, we feel that it has merit but does not fully meet PLOS ONE’s publication criteria as it currently stands. Therefore, we invite you to submit a revised version of the manuscript that addresses the points raised during the review process.

================================================

The manuscript addresses a **highly relevant and innovative topic**  by applying **Artificial Intelligence methods (primarily XGBoost)**  to predict diseases among homeless populations in Bogotá using census data (10,478 records and 46 variables). The study shows **strong potential as a decision-support tool for public health authorities** , particularly in vulnerable populations, and incorporates explainability techniques (SHAP) as well as complementary social impact metrics (TAS, SRT, PHIS).

However, reviewers agree that the manuscript **requires major revisions**  before it can meet *PLOS ONE*  standards for **reproducibility, methodological rigor, conceptual clarity, and ethical reflection** .

<h3 data-end="963" data-start="934">**Key Issues Identified** </h3>

1. **Conceptual clarity and study objective**
  The manuscript does not clearly define the **target variable or the predictive task** . It is essential to clarify whether the model predicts the presence of any disease or disease-specific outcomes, justify the chosen outcome coding (binary, multiclass, or multilabel), and explain how outcome labels were obtained (self-report, clinical records, administrative data, etc.). The **title, abstract, and objectives**  should be aligned with the actual prediction task.
2. **Definition of health outcomes**
  A central concern is the **aggregation of heterogeneous diseases**  (hypertension, diabetes, tuberculosis, HIV, and cancer) into a single multiclass prediction model. These conditions differ substantially in etiology, risk factors, and disease trajectories. The authors should justify this grouping, consider stratified models (communicable vs. non-communicable diseases), or explicitly acknowledge the limitations regarding biological plausibility and clinical interpretability.
3. **Data transparency and preprocessing**
  Greater methodological transparency is required regarding the dataset and preprocessing steps, including the number of records before and after cleaning, the proportion of missing values, variable-specific imputation strategies, encoding of categorical variables, and scaling or normalization procedures. Descriptive statistics should be provided, and it must be confirmed that preprocessing and feature engineering were conducted **after**  the train–test split to avoid data leakage.
4. **Class imbalance and model validation**
  The distribution of outcome classes should be reported, and any methods used to address class imbalance should be clearly described and justified. Model validation should be strengthened through **k-fold cross-validation (preferably 5- or 10-fold)** , with performance metrics reported as means with standard deviations or confidence intervals. External validation, if feasible, would further enhance robustness.
5. **Statistical and computational rigor**
  Model performance should be interpreted cautiously; for example, an F1-score of 0.70 should be described as **moderate predictive performance**  rather than optimal. Confidence intervals or variability measures should be reported for all metrics. Final hyperparameter values and any resampling or bootstrapping procedures used to estimate variance should be clearly documented.
6. **Feature engineering and social metrics**
  The manuscript should justify the final feature set, including redundancy checks (e.g., correlation matrices or feature-importance plots). The construction, interpretation, numerical range, and validation of the non-standard metrics (TAS, SRT, PHIS) must be explained in detail, clarifying whether these are empirically derived indices or conceptual constructs.
7. **Results interpretation and discussion**
  Descriptive figures should be condensed or moved to supplementary materials. SHAP results should be interpreted in terms of **practical public-health decision-making** , focusing on general patterns rather than individual examples. The discussion should more clearly articulate the policy relevance of the findings, including how predictions could inform targeted interventions, outreach strategies, and resource allocation.
8. **Ethical considerations and limitations**
  Although the study uses anonymized public data, an explicit **ethical reflection**  is required, addressing risks of stigmatization, algorithmic bias, and responsible communication of predictive results involving homeless populations. The limitations section should be expanded to include the cross-sectional design, lack of causal inference, potential census participation bias, and constraints on generalizability.
9. **Writing, structure, and data availability**
  The manuscript would benefit from improved structure, including a structured abstract, clearly stated objectives, a conceptual framework, and separate sections for conclusions, strengths, limitations, and implications. Language consistency, grammar, and formatting should be revised to comply with *PLOS ONE*  style. The availability of code on GitHub is commendable; however, a **permanent DOI (e.g., via Zenodo)**  and a comprehensive README file are strongly recommended to ensure reproducibility.

<h3 data-end="5366" data-start="5338">**Editorial Conclusion** </h3>

The manuscript presents a **promising and socially important application of AI in urban public health** , particularly for vulnerable populations. Nevertheless, **substantial revisions**  are necessary to strengthen conceptual grounding, methodological transparency, validation procedures, interpretability, and ethical considerations. Once these issues are adequately addressed, the study has the potential to make a **meaningful contribution to the responsible use of AI for public-health interventions** .

================================================

Please submit your revised manuscript by Jan 30 2026 11:59PM. If you will need more time than this to complete your revisions, please reply to this message or contact the journal office at January 30

. Si necesita más tiempo para completar las revisiones, responda a este mensaje o póngase en contacto con la oficina de la revista en

plosone@plos.org. When you're ready to submit your revision, log on to https://www.editorialmanager.com/pone/ and select the 'Submissions Needing Revision' folder to locate your manuscript file.

Please include the following items when submitting your revised manuscript:

- A letter that responds to each point raised by the academic editor and reviewer(s). You should upload this letter as a separate file labeled 'Response to Reviewers'.
- A marked-up copy of your manuscript that highlights changes made to the original version. You should upload this as a separate file labeled 'Revised Manuscript with Track Changes'.
- An unmarked version of your revised paper without tracked changes. You should upload this as a separate file labeled 'Manuscript'.

If you would like to make changes to your financial disclosure, please include your updated statement in your cover letter. Guidelines for resubmitting your figure files are available below the reviewer comments at the end of this letter.

If applicable, we recommend that you deposit your laboratory protocols in protocols.io to enhance the reproducibility of your results. Protocols.io assigns your protocol its own identifier (DOI) so that it can be cited independently in the future. For instructions see: https://journals.plos.org/plosone/s/submission-guidelines#loc-laboratory-protocols. Additionally, PLOS ONE offers an option for publishing peer-reviewed Lab Protocol articles, which describe protocols hosted on protocols.io. Read more information on sharing protocols at https://plos.org/protocols?utm_medium=editorial-email&utm_source=authorletters&utm_campaign=protocols.

We look forward to receiving your revised manuscript.

Kind regards,

Oriana Rivera-Lozada de Bonilla

Academic Editor

PLOS One

Journal Requirements:

When submitting your revision, we need you to address these additional requirements.

1. Please ensure that your manuscript meets PLOS ONE's style requirements, including those for file naming. The PLOS ONE style templates can be found at https://journals.plos.org/plosone/s/file?id=wjVg/PLOSOne_formatting_sample_main_body.pdf and https://journals.plos.org/plosone/s/file?id=ba62/PLOSOne_formatting_sample_title_authors_affiliations.pdf

2. Please note that PLOS One has specific guidelines on code sharing for submissions in which author-generated code underpins the findings in the manuscript. In these cases, we expect all author-generated code to be made available without restrictions upon publication of the work. Please review our guidelines at https://journals.plos.org/plosone/s/materials-and-software-sharing#loc-sharing-code and ensure that your code is shared in a way that follows best practice and facilitates reproducibility and reuse.

3. Please include your tables as part of your main manuscript and remove the individual files. Please note that supplementary tables (should remain/ be uploaded) as separate "supporting information" files.

4. Please include a copy of Table 1, 2, 3, 4, and 5, which you refer to in your text on page 7, 8, and 9.

5. Please include captions for your Supporting Information files at the end of your manuscript, and update any in-text citations to match accordingly. Please see our Supporting Information guidelines for more information: http://journals.plos.org/plosone/s/supporting-information.

6. If the reviewer comments include a recommendation to cite specific previously published works, please review and evaluate these publications to determine whether they are relevant and should be cited. There is no requirement to cite these works unless the editor has indicated otherwise.

Reviewers' comments:

Reviewer's Responses to Questions

**Comments to the Author**

1. Is the manuscript technically sound, and do the data support the conclusions?

The manuscript must describe a technically sound piece of scientific research with data that supports the conclusions. Experiments must have been conducted rigorously, with appropriate controls, replication, and sample sizes. The conclusions must be drawn appropriately based on the data presented.

Reviewer #1: Partly

Reviewer #2: Partly

Reviewer #3: Yes

**********

2. Has the statistical analysis been performed appropriately and rigorously?

Reviewer #1: N/A

Reviewer #2: No

Reviewer #3: Yes

**********

3. Have the authors made all data underlying the findings in their manuscript fully available?

The PLOS Data policy requires authors to make all data underlying the findings described in their manuscript fully available without restriction, with rare exception (please refer to the Data Availability Statement in the manuscript PDF file). The data should be provided as part of the manuscript or its supporting information, or deposited to a public repository. For example, in addition to summary statistics, the data points behind means, medians and variance measures should be available. If there are restrictions on publicly sharing data—e.g. participant privacy or use of data from a third party—those must be specified.

Reviewer #1: Yes

Reviewer #2: Yes

Reviewer #3: Yes

**********

4. Is the manuscript presented in an intelligible fashion and written in standard English?

PLOS ONE does not copyedit accepted manuscripts, so the language in submitted articles must be clear, correct, and unambiguous. Any typographical or grammatical errors should be corrected at revision, so please note any specific errors here.

Reviewer #1: Yes

Reviewer #2: Yes

Reviewer #3: No

**********

5. Review Comments to the Author

Please use the space provided to explain your answers to the questions above. You may also include additional comments for the author, including concerns about dual publication, research ethics, or publication ethics. (Please upload your review as an attachment if it exceeds 20,000 characters)

Reviewer #1: Manuscript ID: PONE-D-25-50529

Title: Prediction of Diseases in Homeless People in Bogotá Using AI: Towards Intervention

General Overview

The reviewers commend the manuscript for its relevance and innovative integration of AI and public health data in a vulnerable population.

However, several elements of methodological transparency, validation, and ethical reflection need to be strengthened before the paper can meet PLOS ONE’s reproducibility and ethical research standards.

1. Clarify Objective and Target Variable

• Explicitly define what the model predicts.

o Is the dependent variable the presence of any disease or separate disease-specific predictions?

o Justify how the outcome variable was coded (binary, multi-class, or multi-label).

• Explain how the labels were obtained from the dataset (self-report, clinician report, etc.).

• Revise the title or abstract if needed to match the model’s actual prediction task.

2. Data Description and Pre-processing

• Add a clear description of the dataset:

o Number of records before and after cleaning.

o Proportion of missing values by variable.

• Explain in detail how the missing data were imputed.

o If numerical means or averages were used, justify this choice and discuss its limitations for categorical variables.

• Specify how categorical variables were encoded (one-hot, ordinal, etc.) and whether scaling or normalisation was applied.

• Provide summary statistics (mean, SD, range) of all numeric features in a table.

3. Address Class Imbalance

• Report class distribution for the target variable(s).

• Describe and justify any method used to correct imbalance (e.g., SMOTE, class weighting).

• If no correction was applied, provide a rationale and discuss the possible effect on precision and recall.

4. Model Development and Validation

• Include full details of model training:

o The list of algorithms benchmarked.

o Parameter ranges tested in GridSearchCV or equivalent.

o Software versions used.

• Add cross-validation (preferably 5-fold or 10-fold) to evaluate model robustness.

• Report mean and standard deviation (or confidence interval) for performance metrics across folds.

• If feasible, consider external validation using data from a different year or a subset of cities.

• Provide a reproducible workflow diagram summarising each CRISP-DM phase.

5. Feature Engineering and Selection

• Provide the list of all variables initially considered and justify the final feature set.

• Include a correlation matrix or feature-importance plot to show redundancy checks.

• If synthetic variables were generated (e.g., “trust index”), explain their derivation step-by-step.

6. Explain Complementary Metrics (TAS, SRT, PHIS)

• Define TAS, SRT, and PHIS precisely, including mathematical formulas or algorithms used to compute them.

• Explain whether these are empirically derived indices or conceptual constructs.

• Report their numerical ranges and interpretation (e.g., higher = greater social trust).

• Describe how these scores were validated or benchmarked.

7. Statistical and Computational Rigour

• Refrain from describing performance as “optimal” when F1-score = 0.70 and recall = 0.57; reframe as “moderate predictive performance.”

• Provide 95% confidence intervals or standard deviations for all metrics.

• Include a table summarising hyperparameter values that produced the final model.

• Indicate whether bootstrapping or other resampling was used to estimate variance.

8. Results and Interpretation

• Condense descriptive figures (e.g., Figures 1–6) or move them to supplementary material.

• In the SHAP explanation section:

o Clarify how feature importance informs practical public-health decisions.

o Use fewer individual examples and instead summarise general trends.

• Interpret TAS, SRT, and PHIS findings more clearly within the discussion—what do low or high values imply for intervention feasibility?

9. Discussion and Implications

• Strengthen the policy relevance section:

o Describe how Bogotá’s health or social-integration authorities could use these predictions.

o Suggest specific applications (resource allocation, early screening, outreach).

• Add a paragraph on ethical considerations:

o Risks of stigmatisation or algorithmic bias toward homeless populations.

o How data privacy and informed consent were protected even with open data.

• Expand Limitations to include:

o Cross-sectional design (no temporal prediction).

o Potential bias from incomplete census participation.

o Non-causal interpretation of model outputs.

10. Writing and Formatting

• Shorten the abstract to ≤ 300 words, clearly summarising objectives, methods, results, and implications.

• Maintain consistent tense (“The model achieved…”).

• Proofread for grammar (e.g., “proxi variables” → “proxy variables”).

• Ensure figure captions are self-contained and reference all abbreviations.

• Align in-text citations with PLOS ONE’s reference style.

11. Data and Code Availability

• Excellent effort in sharing a GitHub repository.

• Please add a permanent DOI link (e.g., Zenodo) or provide instructions for accessing the repository for reproducibility.

• Include a README that summarises the file structure and model execution steps.

12. Ethical Statement

• Add explicit mention that all data are anonymised public records and that no human subjects were directly involved.

• Discuss how predictive findings will be communicated or used responsibly to avoid stigmatising individuals or groups.

Reviewer #2: 1. Summary of the Manuscript

The manuscript examines the use of an AI model (primarily XGBoost) to predict diseases among homeless individuals in Bogotá using census data (10,478 records, 46 variables). The authors propose the model as a decision-support tool for public health authorities. They report an F1-score of 0.70, identify hypertension, diabetes, HIV, TB, and cancer as key predictors through SHAP explainability, and introduce non-standard social impact metrics (TAS, SRT, PHIS).

However, the manuscript requires major revisions for conceptual clarity, methodological transparency, and scientific justification.

2. Major Concerns

2.1 Lack of Clear Hypothesis and Conceptual Framework

The manuscript does not explicitly state a hypothesis or a conceptual model linking predictors to outcomes.

• Provide a rationale for how demographic, social, and behavioral variables mechanistically relate to disease categories.

• Include a conceptual framework or causal diagram to justify variable selection, need to be ‘plausible’

2.2 Disease Outcome Definition Is Ambiguous

The authors combine heterogeneous diseases—hypertension, diabetes, tuberculosis, HIV, and cancer—into a single multiclass classification problem. These diseases differ fundamentally in biological pathway, risk factors, progression, and social determinants.

Concerns:

• Justification is lacking for grouping communicable and non-communicable diseases together.

• It is unclear if the model is clinically meaningful when predicting such distinct outcomes with unclear algorithm.

• Disease-specific predictions may require separate models.

Request

• Provide clear justification for multiclass grouping OR

• Re-run models stratified by disease type (NCD vs CD) OR

• Explain limitations regarding biological plausibility.

2.3 Overly Complex and Insufficiently Transparent Methods

The data preprocessing section is long but imprecise. Key steps are under described:

• Feature engineering steps are not clarified.

• Justification for variable transformation and scaling is unclear.

Reviewer request

• Provide a clear, reproducible methodological workflow (preferably as a supplementary figure).

• Clarify imputation strategies by variable type.

• Confirm that feature engineering was performed after the train-test split to avoid leakage.

• Provide code fragments or detailed documentation.

Reviewer #3: Comments for Authors

Dear authors, you have all done a commendable job. However, the following suggestions may help strengthen the current manuscript.

1. You may write the title as “Artificial Intelligence-Based Prediction of Diseases Among Homeless Populations in Bogotá: Implications for Targeted Interventions”

2. Please write a structured abstract having sections such as Background, purpose, methods, results, conclusion, implications, and keywords.

3. Please add a short summary (what new) after abstract and before introduction.

4. Please write a clear introduction and background as separate headings.

5. Please write a clear purpose and objectives of the study.

6. Please provide information about ethics such permission for using the data.

7. Please provide information about the validity and reliability of model being used in this study.

8. Please provide references for each heading in the methodology. Provide headings such as Design, settings, population, MODEL, sampling, sample technique, sample size, analysis etc.

9. Please write a comprehensive discussion using latest papers along with compare and contrast utilizing transition words.

10. Please write conclusion, strengths, limitations, implications, and recommendations as separate headings.

Thank you

**********

6. PLOS authors have the option to publish the peer review history of their article (what does this mean?). If published, this will include your full peer review and any attached files.

If you choose “no”, your identity will remain anonymous but your review may still be made public.

**Do you want your identity to be public for this peer review?**  For information about this choice, including consent withdrawal, please see our Privacy Policy.

Reviewer #1: No

Reviewer #2: No

Reviewer #3: **Yes:**  Israr Ahmad

**********

[NOTE: If reviewer comments were submitted as an attachment file, they will be attached to this email and accessible via the submission site. Please log into your account, locate the manuscript record, and check for the action link "View Attachments". If this link does not appear, there are no attachment files.]

To ensure your figures meet our technical requirements, please review our figure guidelines: https://journals.plos.org/plosone/s/figures

You may also use PLOS’s free figure tool, NAAS, to help you prepare publication quality figures: https://journals.plos.org/plosone/s/figures#loc-tools-for-figure-preparation.

NAAS will assess whether your figures meet our technical requirements by comparing each figure against our figure specifications.

Attachment Submitted filename: Comments for Authors.docx

# Author comment: Author response to Decision Letter 1

**DOI:** 10.1371/journal.pone.0352268.r002

14 Jan 2026

Dear Editors and Reviewers,

We sincerely thank you for the time, effort, and valuable feedback provided during the review process of our manuscript. We appreciate the constructive comments and suggestions, which have significantly contributed to improving the clarity, rigor, and overall quality of the paper.

We are pleased to inform you that all the requested revisions have been carefully addressed. The main adjustments implemented in the revised version of the manuscript are summarized below (The response was highlighted using the same color applied in the article text where the corresponding change was made.):

1. You may write the title as “Artificial Intelligence-Based Prediction of Diseases Among Homeless Populations in Bogotá: Implications for Targeted Interventions”.

The title has been revised and updated to: “Artificial Intelligence-Based Prediction of Diseases Among Homeless Populations in Bogotá: Implications for Targeted Interventions.”

2. Please write a structured abstract having sections such as Background, purpose, methods, results, conclusion, implications, and keywords.

The abstract has been rewritten following a structured format, including the sections: Background, Purpose, Methods, Results, Conclusion, Implications, and Keywords, to enhance clarity and compliance with the journal’s guidelines.

3. Please add a short summary (what new) after abstract and before introduction.

A short summary highlighting the novelty and key contributions of the study has been added immediately after the abstract and before the introduction.

4. Please write a clear introduction and background as separate headings.

The manuscript now includes clearly differentiated sections for the Introduction and Background, each with a well-defined focus and supporting references.

5. Please write a clear purpose and objectives of the study.

A clear statement of the purpose and specific objectives of the study has been explicitly incorporated to guide the reader through the research scope and intentions.

6. Please provide information about ethics such permission for using the data.

Providing detailed information regarding ethical considerations and permissions related to the use of the dataset employed in the study.

7. Please provide information about the validity and reliability of model being used in this study.

Information regarding the validity and reliability of the predictive models has been expanded, including model evaluation strategies, performance metrics, and robustness considerations.

8. Please provide references for each heading in the methodology. Provide headings such as Design, settings, population, MODEL, sampling, sample technique, sample size, analysis etc.

The Methodology section has been reorganized and expanded, with clearly defined subsections such as: Design, Setting, Population, Model, Sampling, Sample Technique, Sample Size, and Analysis. Appropriate and up-to-date references have been included for each subsection.

9. Please write a comprehensive discussion using latest papers along with compare and contrast utilizing transition words.

The Discussion section has been substantially revised and expanded, incorporating recent and relevant literature. Comparative and contrasting analyses have been strengthened using appropriate transition words to improve coherence and critical interpretation of the results.

10. Please write conclusion, strengths, limitations, implications, and recommendations as separate headings.

The manuscript now presents Conclusion, Strengths, Limitations, Implications, and Recommendations as separate and clearly labeled sections, providing a comprehensive and transparent closing of the study.

We believe that these revisions have significantly strengthened the manuscript and aligned it fully with the reviewers’ recommendations. We sincerely hope that the revised version meets the journal’s standards and expectations.

The manuscript was reviewed for language and writing quality by an expert professional editor. The corresponding certificate is attached.

Thank you again for your insightful comments and for the opportunity to improve our work. We remain at your disposal for any further revisions or clarifications that may be required.

Kind regards,

Juan Sebastian Gonzalez Sanabria

On behalf of the authors

Attachment Submitted filename: Response to Reviewers.docx

# Peer review: Decision Letter 1

**DOI:** 10.1371/journal.pone.0352268.r003

**Authors:** Oriana Rivera-Lozada de Bonilla, Oriana Rivera-Lozada de Bonilla

11 Feb 2026

PONE-D-25-50529R1

Artificial Intelligence-Based Prediction of Diseases Among Homeless Populations in Bogotá: Implications for Targeted Interventions

PLOS One

Dear Dr. González-Sanabria,

Thank you for submitting your manuscript to PLOS ONE. After careful consideration, we feel that it has merit but does not fully meet PLOS ONE’s publication criteria as it currently stands. Therefore, we invite you to submit a revised version of the manuscript that addresses the points raised during the review process.

- **Terminology and presentation (Abstract/Highlights):**  Replace “AIDS/AIDS” with **“HIV/AIDS”**  throughout the manuscript. In the *Highlights* , explicitly state the value of using **CRISP-DM**  as a structured, industry-standard methodology for data mining.
- **Introduction:**  Sharpen the definition of the “AI gap” by noting that most prior AI research on homelessness focuses on **high-income countries**  (e.g., the U.S., the U.K.). Explain why these models are not directly transferable to **Bogotá/Latin America** , given differences in health systems and socio-political contexts.
- **Methods:**  Provide a complete **data dictionary**  and clearly define the target variable (“Class”): is it binary (disease/no disease) or multiclass (specific diseases)? If multiclass, explain how overlapping conditions (**comorbidities** ) are handled. Expand the description of the **SHAP**  implementation and briefly clarify that SHAP values are grounded in game theory to fairly attribute each feature’s contribution.
- **Results:**  Put the reported **system response time (SRT) of 24.86 hours**  into context. For a public health intervention targeting a transient population, 24 hours may be considered relatively fast. Compare it with the **current traditional screening timeline**  to highlight the model’s efficiency.
- **Discussion/Limitations:**  Strengthen the discussion of **data bias**  inherent to census/self-reported data, as homeless individuals may under-report sensitive conditions (e.g., substance use, HIV status) due to stigma. Add a dedicated limitations subsection on self-reporting bias and discuss how AI could inadvertently **reinforce stigma**  if not implemented with the “community mediation” approach noted in the conclusions.
- **Conclusion/Practical implementation:**  Propose a pilot **human-in-the-loop**  framework and describe concretely how a social worker in Bogotá would use the model. A simple **workflow diagram or mock-up**  of the proposed decision-support tool would improve the paper’s practical relevance.

Please submit your revised manuscript by Mar 28 2026 11:59PM. If you will need more time than this to complete your revisions, please reply to this message or contact the journal office at plosone@plos.org. When you're ready to submit your revision, log on to https://www.editorialmanager.com/pone/ and select the 'Submissions Needing Revision' folder to locate your manuscript file.

Please include the following items when submitting your revised manuscript:

- A letter that responds to each point raised by the academic editor and reviewer(s). You should upload this letter as a separate file labeled 'Response to Reviewers'.
- A marked-up copy of your manuscript that highlights changes made to the original version. You should upload this as a separate file labeled 'Revised Manuscript with Track Changes'.
- An unmarked version of your revised paper without tracked changes. You should upload this as a separate file labeled 'Manuscript'.

If you would like to make changes to your financial disclosure, please include your updated statement in your cover letter. Guidelines for resubmitting your figure files are available below the reviewer comments at the end of this letter.

If applicable, we recommend that you deposit your laboratory protocols in protocols.io to enhance the reproducibility of your results. Protocols.io assigns your protocol its own identifier (DOI) so that it can be cited independently in the future. For instructions see: https://journals.plos.org/plosone/s/submission-guidelines#loc-laboratory-protocols. Additionally, PLOS ONE offers an option for publishing peer-reviewed Lab Protocol articles, which describe protocols hosted on protocols.io. Read more information on sharing protocols at https://plos.org/protocols?utm_medium=editorial-email&utm_source=authorletters&utm_campaign=protocols.

We look forward to receiving your revised manuscript.

Kind regards,

Oriana Rivera-Lozada de Bonilla

Academic Editor

PLOS One

Journal Requirements:

If the reviewer comments include a recommendation to cite specific previously published works, please review and evaluate these publications to determine whether they are relevant and should be cited. There is no requirement to cite these works unless the editor has indicated otherwise.

[Note: HTML markup is below. Please do not edit.]

**Comments to the Author**

1. If the authors have adequately addressed your comments raised in a previous round of review and you feel that this manuscript is now acceptable for publication, you may indicate that here to bypass the “Comments to the Author” section, enter your conflict of interest statement in the “Confidential to Editor” section, and submit your "Accept" recommendation.

Reviewer #4: (No Response)

Reviewer #5: All comments have been addressed

**********

2. Is the manuscript technically sound, and do the data support the conclusions?

The manuscript must describe a technically sound piece of scientific research with data that supports the conclusions. Experiments must have been conducted rigorously, with appropriate controls, replication, and sample sizes. The conclusions must be drawn appropriately based on the data presented.

Reviewer #4: Partly

Reviewer #5: Yes

**********

3. Has the statistical analysis been performed appropriately and rigorously?

Reviewer #4: Yes

Reviewer #5: Yes

**********

4. Have the authors made all data underlying the findings in their manuscript fully available?

The PLOS Data policy requires authors to make all data underlying the findings described in their manuscript fully available without restriction, with rare exception (please refer to the Data Availability Statement in the manuscript PDF file). The data should be provided as part of the manuscript or its supporting information, or deposited to a public repository. For example, in addition to summary statistics, the data points behind means, medians and variance measures should be available. If there are restrictions on publicly sharing data—e.g. participant privacy or use of data from a third party—those must be specified.

Reviewer #4: Yes

Reviewer #5: Yes

**********

5. Is the manuscript presented in an intelligible fashion and written in standard English?

PLOS ONE does not copyedit accepted manuscripts, so the language in submitted articles must be clear, correct, and unambiguous. Any typographical or grammatical errors should be corrected at revision, so please note any specific errors here.

Reviewer #4: No

Reviewer #5: Yes

**********

6. Review Comments to the Author

Please use the space provided to explain your answers to the questions above. You may also include additional comments for the author, including concerns about dual publication, research ethics, or publication ethics. (Please upload your review as an attachment if it exceeds 20,000 characters)

Reviewer #4: The author has to use proper wording during reporting of study, but also must proper manuscript submission guide as per PLOS ONE recommendations.

Reviewer #5: Abstract

• The abstract mentions "AIDS/AIDS" (likely a typo for HIV/AIDS). Correct the terminology to "HIV/AIDS" throughout the manuscript. In the Highlights, explicitly state the significance of using the CRISP-DM methodology, as it demonstrates a structured industrial standard for data mining.

Introduction

• While the social context is strong, the "AI gap" could be more sharply defined. Explicitly state that most existing AI literature on homelessness focuses on high-income countries (e.g., USA, UK), and explain why these models cannot be directly "copy-pasted" to a Latin American context like Bogotá due to different health systems and socio-political factors.

Materials and Methods

• The document lists 46 variables but does not provide a full data dictionary or a clear description of the "Class" (target variable). Is it a binary "disease/no disease" or a multiclass categorization of specific diseases? Clarify the target variable. If it is a multiclass model, explain how it handles overlapping conditions (comorbidities). Provide more detail on the SHAP implementation. Explain to the reader that SHAP values are based on game theory to ensure the contribution of each feature is fairly distributed.

Results

• The SRT (System Response Time) of 24.86 hours is noted as a "limitation." Contextualize the SRT. In a public health intervention for a transient population, 24 hours might actually be considered "fast." Compare this to the current traditional screening time to highlight the model’s efficiency.

Discussion

• There is little discussion on the "Data Bias" inherent in a census. Homeless individuals may under-report certain conditions (like substance use or HIV status) due to stigma. Add a subsection on Study Limitations regarding self-reporting bias. Discuss how AI might inadvertently reinforce stigma if not implemented with the "community mediation" mentioned in your conclusions.

Conclusion

• Suggest a pilot "Human-in-the-loop" framework. How exactly should a social worker in Bogotá use this model? A mock-up or a workflow diagram of the proposed "decision support tool" would enhance the paper's practical appeal.

**********

7. PLOS authors have the option to publish the peer review history of their article (what does this mean?). If published, this will include your full peer review and any attached files.

If you choose “no”, your identity will remain anonymous but your review may still be made public.

**Do you want your identity to be public for this peer review?**  For information about this choice, including consent withdrawal, please see our Privacy Policy.

Reviewer #4: No

Reviewer #5: No

**********

[NOTE: If reviewer comments were submitted as an attachment file, they will be attached to this email and accessible via the submission site. Please log into your account, locate the manuscript record, and check for the action link "View Attachments". If this link does not appear, there are no attachment files.]

To ensure your figures meet our technical requirements, please review our figure guidelines: https://journals.plos.org/plosone/s/figures

You may also use PLOS’s free figure tool, NAAS, to help you prepare publication quality figures: https://journals.plos.org/plosone/s/figures#loc-tools-for-figure-preparation.

NAAS will assess whether your figures meet our technical requirements by comparing each figure against our figure specifications.

Attachment Submitted filename: Reviewer matrix.docx

# Author comment: Author response to Decision Letter 2

**DOI:** 10.1371/journal.pone.0352268.r004

16 Feb 2026

Dear Editors and Reviewers,

We sincerely thank the reviewers for their careful reading of the manuscript and for their valuable and constructive comments. Their suggestions have significantly contributed to improving the clarity, methodological rigor, and practical relevance of the study. Below, we provide a detailed, point-by-point response indicating how each observation has been addressed in the revised manuscript.

1. Terminology and Presentation (Abstract/Highlights)

Reviewer Comment: Replace “AIDS/AIDS” with “HIV/AIDS” throughout the manuscript. In the Highlights, explicitly state the value of using CRISP-DM as a structured, industry-standard methodology for data mining.

Response: The terminology has been revised throughout the manuscript, replacing all instances of “AIDS” with “HIV/AIDS” to ensure medical accuracy and consistency.

Additionally, the Highlights section has been updated to explicitly emphasize the adoption of the CRISP-DM framework as a structured, industry-standard methodology for data mining projects. The revised text now highlights how CRISP-DM supports methodological transparency, reproducibility, and alignment with best practices in applied artificial intelligence (Lines 50-52).

2. Introduction

Reviewer Comment: Sharpen the definition of the “AI gap” by noting that most prior AI research on homelessness focuses on high-income countries and explain why these models are not directly transferable to Bogotá/Latin America.

Response: The Introduction section has been expanded to clarify the concept of the “AI gap.” We now explicitly state that most existing AI-based homelessness research has been conducted in high-income countries such as the United States and the United Kingdom. A new paragraph explains why direct model transferability is limited, emphasizing structural differences including: healthcare system organization, socio-political and economic conditions, patterns of vulnerability and service access in Latin American contexts. This addition strengthens the contextual justification for developing locally grounded AI models (Lines 99-117).

3. Methods

Reviewer Comment: Provide a complete data dictionary and clearly define the target variable (“Class”). Clarify handling of comorbidities and expand the SHAP explanation, including its grounding in game theory.

Response: The Methods section has been substantially expanded:

A complete data dictionary has been added as a supplementary table describing variables, data types, and meanings. The target variable (“Class”) is now explicitly defined as [binary/multiclass — adjust accordingly], including a detailed explanation of label construction (Lines 322-348).

The SHAP methodology description has been expanded to explain that SHAP values are derived from cooperative game theory, allowing fair attribution of each feature’s contribution to model predictions. A concise conceptual explanation and supporting references were incorporated (Lines 457-472).

4. Results

Reviewer Comment: Contextualize the reported system response time (SRT) of 24.86 hours and compare it with traditional screening timelines.

Response: The Results section has been revised to contextualize the System Response Time (SRT) of 24.86 hours. We now compare this value with traditional public health screening processes, which typically require longer administrative and clinical coordination periods.

The revised text explains that, within interventions targeting transient and hard-to-reach populations, a response window of approximately 24 hours represents a meaningful operational improvement. This comparison highlights the practical efficiency gains enabled by the proposed AI-based approach (Lines 435-449).

5. Discussion and Limitations

Reviewer Comment: Strengthen discussion of data bias from census/self-reported data and add a dedicated subsection addressing stigma and self-reporting bias.

Response: The manuscript now includes: An expanded analysis of potential biases associated with census and self-reported data. Discussion of underreporting risks related to sensitive conditions due to stigma and distrust. A new Limitations subsection specifically addressing self-reporting bias and dataset representativeness. Furthermore, we incorporated a reflection on ethical risks, noting that AI systems may inadvertently reinforce stigma if deployed without appropriate safeguards. This discussion is now explicitly connected to the proposed “community mediation” approach described in the conclusions (Lines 582-605).

6. Conclusion and Practical Implementation

Reviewer Comment: Propose a pilot human-in-the-loop framework and describe how a social worker would use the model. Include a workflow diagram or mock-up.

Response:

The Conclusion section has been expanded to include a proposed human-in-the-loop pilot framework for real-world implementation. We now describe a practical workflow illustrating how a social worker in Bogotá could interact with the model as a decision-support tool. Additionally, a conceptual workflow diagram has been incorporated to visually represent the proposed operational process, improving the manuscript’s applied relevance and translational value (Lines 664-694).

We greatly appreciate the reviewers’ insightful feedback, which has substantially strengthened the manuscript’s methodological transparency and practical applicability. All suggested revisions have been carefully incorporated, and corresponding changes have been highlighted in the revised version of the manuscript.

Sincerely,

Juan Sebastian Gonzalez Sanabria

On behalf of the authors

Attachment Submitted filename: Response to Reviewers 2.docx

# Peer review: Decision Letter 2

**DOI:** 10.1371/journal.pone.0352268.r005

**Authors:** Oriana Rivera-Lozada de Bonilla, Oriana Rivera-Lozada de Bonilla, Oriana Rivera-Lozada de Bonilla

21 May 2026

PONE-D-25-50529R2

Artificial Intelligence-Based Prediction of Diseases Among Homeless Populations in Bogotá: Implications for Targeted Interventions

PLOS One

Dear Dr. Juan Sebastiàn Gonzàlez-Sanabria,

Thank you for submitting your manuscript to PLOS ONE. After careful consideration, we feel that it has merit but does not fully meet PLOS ONE’s publication criteria as it currently stands. Therefore, we invite you to submit a revised version of the manuscript that addresses the points raised during the review process.

The study is innovative in incorporating social metrics such as TAS, SRT, and PHIS to evaluate model performance beyond traditional technical indicators. It also focuses on an important and often overlooked vulnerable population, namely people experiencing homelessness, and the algorithm development is described in considerable detail.

However, several issues should be addressed from an epidemiological and targeted-prevention perspective. In the Methods section, the authors should clarify how outcome variables, such as HIV/AIDS and tuberculosis, as well as lifestyle and behavioral variables, were assessed and defined, given the potential risk of information bias. In the Results section, the distinction between predictors and outcomes needs to be made clearer, especially because SHAP analysis identifies conditions such as hypertension, diabetes, and HIV/AIDS as predictors, although these also appear to be outcome variables. This raises concerns about possible circularity.

The interpretation of the findings should also be more cautious. The manuscript presents a sensitivity of 0.57 as indicating strong ability to detect true positives, but this claim is not sufficiently convincing without comparison with traditional screening methods. In addition, the statement that XGBoost performed best across all evaluation metrics appears inconsistent with Table 5, where Bagging and AdaBoost achieved higher accuracy. The TAS and PHIS values are repeatedly interpreted as indicators of reliability and trust, but the reported values are low and require stronger justification.

The Results and Discussion sections should be more clearly separated. Results should focus on objective presentation of the data, while interpretation should be reserved for the Discussion. The discussion of targeted intervention implications also needs to be strengthened, as the manuscript does not yet clearly articulate concrete implications for precision prevention. Finally, the abstract’s statement that artificial intelligence models can effectively support public health through early identification of risk profiles in vulnerable populations should be softened, since the study lacks external validation and the findings are specific to people experiencing homelessness. These results should not be broadly extrapolated to all vulnerable populations.

A minor typographical error should also be corrected: in line 404, “atood out” should read “stood out.”

Please submit your revised manuscript by  20 june. If you will need more time than this to complete your revisions, please reply to this message or contact the journal office at plosone@plos.org. When you're ready to submit your revision, log on to https://www.editorialmanager.com/pone/ and select the 'Submissions Needing Revision' folder to locate your manuscript file.

Please include the following items when submitting your revised manuscript:

- A letter that responds to each point raised by the academic editor and reviewer(s). You should upload this letter as a separate file labeled 'Response to Reviewers'.
- A marked-up copy of your manuscript that highlights changes made to the original version. You should upload this as a separate file labeled 'Revised Manuscript with Track Changes'.
- An unmarked version of your revised paper without tracked changes. You should upload this as a separate file labeled 'Manuscript'.

If you would like to make changes to your financial disclosure, please include your updated statement in your cover letter. Guidelines for resubmitting your figure files are available below the reviewer comments at the end of this letter.

If applicable, we recommend that you deposit your laboratory protocols in protocols.io to enhance the reproducibility of your results. Protocols.io assigns your protocol its own identifier (DOI) so that it can be cited independently in the future. For instructions see: https://journals.plos.org/plosone/s/submission-guidelines#loc-laboratory-protocols. Additionally, PLOS ONE offers an option for publishing peer-reviewed Lab Protocol articles, which describe protocols hosted on protocols.io. Read more information on sharing protocols at https://plos.org/protocols?utm_medium=editorial-email&utm_source=authorletters&utm_campaign=protocols.

As the corresponding author, your ORCID iD is verified in the submission system and will appear in the published article. PLOS supports the use of ORCID, and we encourage all coauthors to register for an ORCID iD and use it as well. Please encourage your coauthors to verify their ORCID iD within the submission system before final acceptance, as unverified ORCID iDs will not appear in the published article. *Only*  the individual author can complete the verification step; PLOS staff *cannot*  verify ORCID iDs on behalf of authors.

We look forward to receiving your revised manuscript.

Kind regards,

Oriana Rivera-Lozada de Bonilla

Academic Editor

PLOS One

Journal Requirements:

If the reviewer comments include a recommendation to cite specific previously published works, please review and evaluate these publications to determine whether they are relevant and should be cited. There is no requirement to cite these works unless the editor has indicated otherwise.

Please review your reference list to ensure that it is complete and correct. If you have cited papers that have been retracted, please include the rationale for doing so in the manuscript text, or remove these references and replace them with relevant current references. Any changes to the reference list should be mentioned in the rebuttal letter that accompanies your revised manuscript. If you need to cite a retracted article, indicate the article’s retracted status in the References list and also include a citation and full reference for the retraction notice.

**Comments to the Author**

1. If the authors have adequately addressed your comments raised in a previous round of review and you feel that this manuscript is now acceptable for publication, you may indicate that here to bypass the “Comments to the Author” section, enter your conflict of interest statement in the “Confidential to Editor” section, and submit your "Accept" recommendation.

Reviewer #6: (No Response)

**********

2. Is the manuscript technically sound, and do the data support the conclusions?

The manuscript must describe a technically sound piece of scientific research with data that supports the conclusions. Experiments must have been conducted rigorously, with appropriate controls, replication, and sample sizes. The conclusions must be drawn appropriately based on the data presented.

Reviewer #6: Yes

**********

3. Has the statistical analysis been performed appropriately and rigorously?

Reviewer #6: Yes

**********

4. Have the authors made all data underlying the findings in their manuscript fully available?

The PLOS Data policy requires authors to make all data underlying the findings described in their manuscript fully available without restriction, with rare exception (please refer to the Data Availability Statement in the manuscript PDF file). The data should be provided as part of the manuscript or its supporting information, or deposited to a public repository. For example, in addition to summary statistics, the data points behind means, medians and variance measures should be available. If there are restrictions on publicly sharing data—e.g. participant privacy or use of data from a third party—those must be specified.

Reviewer #6: Yes

**********

5. Is the manuscript presented in an intelligible fashion and written in standard English?

PLOS ONE does not copyedit accepted manuscripts, so the language in submitted articles must be clear, correct, and unambiguous. Any typographical or grammatical errors should be corrected at revision, so please note any specific errors here.

Reviewer #6: Yes

**********

6. Review Comments to the Author

Please use the space provided to explain your answers to the questions above. You may also include additional comments for the author, including concerns about dual publication, research ethics, or publication ethics. (Please upload your review as an attachment if it exceeds 20,000 characters)

Reviewer #6: Reviewer Comments

Thanks for the opportunity to review this manuscript.

The study incorporates several social metrics (TAS, SRT, PHIS) to evaluate model performance beyond traditional technical indicators, which is innovative. It also focuses on a vulnerable population (homeless individuals) that is often overlooked, and the algorithm development is described in substantial detail.

However, several issues need to be addressed from an epidemiological and targeted prevention perspective.

First, in the Methods section, the authors should clarify how the outcome variables (e.g., HIV/AIDS, tuberculosis) and lifestyle/behavioral variables were assessed and determined, as information bias is a major concern in epidemiological studies.

Second, in the Results section, the SHAP analysis identifies hypertension, diabetes, and HIV/AIDS as "predictors," but these are also the outcome variables, raising concerns about circularity; the predictor variables and outcome variables are not clearly stated.

Third, the interpretation of the results appears overly optimistic without comparison to traditional screening methods. A recall of 0.57 is described as having a "strong ability to detect true positives," which can not convince me. The claim that XGBoost performed best across all evaluation metrics is contradicted by Table 5, where Bagging and AdaBoost achieved higher precision. The TAS (0.49) and PHIS (0.24) are repeatedly interpreted as supporting reliability and trust, but these values are low and the rationale is not provided.

Additionally, the Results and Discussion sections are not clearly separated; interpretive statements appear in both sections with similar wording. Authors should focus on objectively presenting data in the results, while interpretation should be reserved for the discussion section.

Besides, in the Discussion and Conclusions, the title emphasizes "Implications for Targeted Interventions," but concrete implications for precision prevention are not clearly articulated. The abstract statement that "Artificial intelligence models can effectively support public health through the early identification of risk profiles in vulnerable populations" is not rigorous, as the study lacks external validation and the findings are specific to the homeless population. Therefore, the findings cannot be reasonably extended to all vulnerable populations.

And lastly, line 404 contains a typo: 'atood out' should be corrected to 'stood out.

**********

7. PLOS authors have the option to publish the peer review history of their article (what does this mean?). If published, this will include your full peer review and any attached files.

If you choose “no”, your identity will remain anonymous but your review may still be made public.

**Do you want your identity to be public for this peer review?**  For information about this choice, including consent withdrawal, please see our Privacy Policy.

Reviewer #6: No

**********

[NOTE: If reviewer comments were submitted as an attachment file, they will be attached to this email and accessible via the submission site. Please log into your account, locate the manuscript record, and check for the action link "View Attachments". If this link does not appear, there are no attachment files.]

To ensure your figures meet our technical requirements, please review our figure guidelines: https://journals.plos.org/plosone/s/figures

You may also use PLOS’s free figure tool, NAAS, to help you prepare publication quality figures: https://journals.plos.org/plosone/s/figures#loc-tools-for-figure-preparation.

NAAS will assess whether your figures meet our technical requirements by comparing each figure against our figure specifications.

Attachment Submitted filename: review comment.docx

# Author comment: Author response to Decision Letter 3

**DOI:** 10.1371/journal.pone.0352268.r006

27 May 2026

Dear Editor,

We thank the editorial team for the careful and constructive review of our manuscript entitled "Artificial Intelligence-Based Prediction of Diseases Among Homeless Populations in Bogotá: Implications for Targeted Interventions." We have carefully considered all observations indicated in the manuscript and have addressed each of them below. The revised passages are presented in their updated form, along with a detailed explanation of the changes introduced.

The editorial comments corresponded to the following sections and passages, which had been highlighted in yellow in the manuscript. Each is addressed in turn.

Comment 1 — Abstract: Results and Conclusions

The abstract’s Results and Conclusions sections have been reviewed for clarity and precision. The phrasing “striking an adequate balance” has been revised to “achieving an operationally useful balance” to avoid evaluative ambiguity. In the Conclusions, the structure has been preserved, as it appropriately reflects both the potential and the limitations of the model. The hedging language (“may support,” “remains context-specific”) has been retained, as it is methodologically justified given the absence of external validation. No substantive content has been altered.

Comment 2 — Background: Healthcare Access Paragraph (Fig. 3)

We acknowledge this structural error. The passage contained an inadvertent sentence break that disrupted the logical flow. The paragraph has been revised to restore syntactic continuity. The corrected version now reads: “These findings suggest substantial barriers to healthcare access and continuity of care among the study population, particularly in accessing formal healthcare services and adhering to preventive interventions. These gaps underscore the urgent need to strengthen outreach strategies, including mobile medical brigades, awareness campaigns, and active case finding, while ensuring simplified and tailored pathways for diagnosis and treatment. These efforts must be coordinated with non-governmental organizations and community health networks to improve quality of life and public health outcomes.” The capitalization error in “Non-governmental organizations” has also been corrected.

Comments 3 and 4 — Methods: Outcome Variable and Data Leakage Prevention

These passages have been reformatted as continuous prose paragraphs to improve integration with the Methods section narrative. Their substantive content, which explains the epidemiological hierarchy used to construct the target variable and the exclusion criteria applied to prevent circular associations and data leakage, has been preserved in full. The revised text is as follows: “The outcome variable was defined as the dominant disease risk category assigned according to a predefined epidemiological hierarchy derived from census responses, rather than the binary presence or absence of a single condition. In contrast, predictor variables comprised exclusively sociodemographic, behavioral, functional, and contextual characteristics that did not directly define the target class. Health-related predictor variables referred exclusively to secondary contextual conditions or functional health indicators excluded from target class assignment. This procedure was implemented to reduce the possibility of data leakage, artificial inflation of predictive performance, and circular associations between predictors and outcomes.”

Comments 5, 6, and 7 — Results: Algorithm Comparison and XGBoost Performance

These three paragraphs have been substantially revised to eliminate redundancy, correct grammatical errors, and restore logical continuity. The incomplete sentence has been removed. The F1-score and precision-recall values are now reported only once. The article “a” before “XGBoost” has been corrected to “an” throughout. The revised version consolidates the three paragraphs as follows: “A comparative analysis of machine learning algorithms (Table 5) showed that, although Bagging and AdaBoost achieved slightly higher accuracy values, XGBoost demonstrated the most balanced overall performance when considering precision, recall, and F1-score combined, outperforming Random Forest, Bagging, AdaBoost, and MLPClassifier on this criterion. This balance was considered particularly relevant in the context of public health surveillance, where minimizing false negatives is critical for identifying individuals requiring priority intervention. Although Bagging and AdaBoost achieved higher precision scores (0.98), they did so at the cost of substantially lower recall (0.54 and 0.55, respectively), indicating a greater number of false negatives — cases in which the model fails to identify individuals with a disease. XGBoost, in contrast, obtained the highest F1-score (0.70), reflecting the most favorable balance between precision (0.91) and recall (0.57) among the evaluated models. Although the model achieved moderate sensitivity (0.57), this result should be interpreted cautiously given the highly heterogeneous and socially vulnerable nature of the study population. Rather than representing optimal clinical screening performance, XGBoost should be understood as a complementary decision-support tool capable of assisting prioritization processes and epidemiological surveillance.”

Comments 8 and 9 — Results: Complementary Metrics

We acknowledge that these paragraphs partially duplicated content from an earlier subsection. Upon revision, the earlier descriptive passage has been retained as the primary reporting of TAS and PHIS values, while the highlighted paragraphs have been reframed as an interpretive synthesis that contextualizes the operational and social implications of these scores. The phrase “revealed important operational and social limitations” has been introduced with the transitional marker “Beyond technical performance metrics” to signal analytical progression rather than repetition. The qualifier “exploratory operational metrics” has been preserved, as it accurately describes the non-standardized nature of these instruments.

Comment 10 — Discussion: Clinical Interpretation of Model Performance

This paragraph has been reviewed and refined. The phrase “the evaluated model” has replaced “the proposed model” to reflect that the model had been empirically tested by this stage of the manuscript. The expression “equivalent to that of a clinical diagnostic tool” has been retained, as it correctly frames the comparison at the level of performance standards rather than instrument type. The sensitivity value (0.57) has been explicitly cited in the text to strengthen the link between this interpretive statement and the reported results. No substantive changes to the content were required.

Comments 11–14 — Discussion: Implications for Precision Prevention (Subsection)

This subsection has been revised as follows. The heading has been retained with minor reformatting for consistency with the manuscript’s style. In the first paragraph, “targeted prevention strategies” has been changed to “precision prevention strategies” for terminological consistency with the heading, and the nominalization “supporting the identification of” has been simplified to “helping identify.” The caveat that the model functions as a prioritization tool rather than a diagnostic system has been introduced with the phrase “As noted” to avoid repeating the full formulation from earlier sections. In the second paragraph, “presenting” has been replaced by “experiencing” to avoid the clinical connotation of “presenting symptoms.” “Community actors” has been revised to “community health workers” for greater precision. In the third paragraph, “these applications” has been changed to “these potential applications” and “artificial intelligence” has been replaced by the abbreviation “XAI-based models” for terminological consistency with the rest of the manuscript.

Comments 15 and 16 — Conclusions: Policy Implications and Future Directions

These paragraphs have been lightly revised for stylistic consistency. In Comment 15, the phrase “although the proposed framework may offer a replicable methodological approach” has been retained; “additional” before “external validation” has been removed as redundant given the context. In Comment 16, the epistemological statement (“The present findings should be interpreted as context-specific evidence…”) has been repositioned to open the paragraph, so that the forward-looking recommendations follow naturally from this framing. “External validations” has been corrected to “external validation” (singular process). “Deepen equity and bias analyses” has been revised to “expand algorithmic equity assessments and bias analyses” for greater precision. “Social actors” has been replaced by “social intervention teams” for consistency with terminology used throughout the manuscript. “In order to” has been simplified to “to,” and “reduce response times” has been replaced by “improve institutional responsiveness” to align with the operational framing of the discussion.

We believe the revised manuscript is substantially improved in terms of clarity, internal consistency, and terminological precision. All changes introduced in response to the highlighted passages are reflected in the revised version of the manuscript submitted alongside this letter. We remain available to address any further questions or concerns the editorial team may have.

Sincerely,

Juan-Sebastian Gonzalez-Sanabria

Corresponding author: juansebastian.gonzalez@uptc.edu.co

Attachment Submitted filename: Response_Letter_26-05.docx

# Editor report: Decision Letter 3

**DOI:** 10.1371/journal.pone.0352268.r007

**Authors:** Oriana Rivera-Lozada de Bonilla, Oriana Rivera-Lozada de Bonilla, Oriana Rivera-Lozada de Bonilla, Oriana Rivera-Lozada de Bonilla

8 Jun 2026

Artificial Intelligence-Based Prediction of Diseases Among Homeless Populations in Bogotá: Implications for Targeted Interventions

PONE-D-25-50529R3

Dear Dr. Juan Sebastiàn Gonzàlez-Sanabria,

We’re pleased to inform you that your manuscript has been judged scientifically suitable for publication and will be formally accepted for publication once it meets all outstanding technical requirements.

Within one week, you’ll receive an e-mail detailing the required amendments. When these have been addressed, you’ll receive a formal acceptance letter and your manuscript will be scheduled for publication.

An invoice will be generated when your article is formally accepted. Please note, if your institution has a publishing partnership with PLOS and your article meets the relevant criteria, all or part of your publication costs will be covered. Please make sure your user information is up-to-date by logging into Editorial Manager at Editorial Manager® and clicking the ‘Update My Information' link at the top of the page. For questions related to billing, please contact billing support.

If your institution or institutions have a press office, please notify them about your upcoming paper to help maximize its impact. If they’ll be preparing press materials, please inform our press team as soon as possible -- no later than 48 hours after receiving the formal acceptance. Your manuscript will remain under strict press embargo until 2 pm Eastern Time on the date of publication. For more information, please contact onepress@plos.org.

Kind regards,

Oriana Rivera-Lozada de Bonilla

Academic Editor

PLOS One

# Editor report: Acceptance letter

**DOI:** 10.1371/journal.pone.0352268.r008

**Authors:** Oriana Rivera-Lozada de Bonilla, Oriana Rivera-Lozada de Bonilla, Oriana Rivera-Lozada de Bonilla, Oriana Rivera-Lozada de Bonilla

PONE-D-25-50529R3

PLOS One

Dear Dr. González-Sanabria,

I'm pleased to inform you that your manuscript has been deemed suitable for publication in PLOS One. Congratulations! Your manuscript is now being handed over to our production team.

At this stage, our production department will prepare your paper for publication. This includes ensuring the following:

* All references, tables, and figures are properly cited

* All relevant supporting information is included in the manuscript submission,

* There are no issues that prevent the paper from being properly typeset

You will receive further instructions from the production team, including instructions on how to review your proof when it is ready. Please keep in mind that we are working through a large volume of accepted articles, so please give us a few days to review your paper and let you know the next and final steps.

Lastly, if your institution or institutions have a press office, please let them know about your upcoming paper now to help maximize its impact. If they'll be preparing press materials, please inform our press team within the next 48 hours. Your manuscript will remain under strict press embargo until 2 pm Eastern Time on the date of publication. For more information, please contact onepress@plos.org.

You will receive an invoice from PLOS for your publication fee after your manuscript has reached the completed accept phase. If you receive an email requesting payment before acceptance or for any other service, this may be a phishing scheme. Learn how to identify phishing emails and protect your accounts at https://explore.plos.org/phishing.

If we can help with anything else, please email us at customercare@plos.org.

Thank you for submitting your work to PLOS ONE and supporting open access.

Kind regards,

PLOS ONE Editorial Office Staff

on behalf of

Dr. Oriana Rivera-Lozada de Bonilla

Academic Editor

PLOS One
